# Cellular dormancy in minimal residual disease following targeted therapy

**DOI:** 10.1186/s13058-021-01416-9

**Published:** 2021-06-04

**Authors:** Jason R. Ruth, Dhruv K. Pant, Tien-chi Pan, Hans E. Seidel, Sanjeethan C. Baksh, Blaine A. Keister, Rita Singh, Christopher J. Sterner, Suzanne J. Bakewell, Susan E. Moody, George K. Belka, Lewis A. Chodosh

**Affiliations:** 1 Department of Cancer Biology, Perelman School of Medicine at the University of Pennsylvania, Philadelphia, PA 19104 USA; 2 2-PREVENT Translational Center of Excellence at the Abramson Cancer Center, Perelman School of Medicine at the University of Pennsylvania, Philadelphia, PA 19104 USA; 3 the Abramson Family Cancer Research Institute, Perelman School of Medicine at the University of Pennsylvania, Philadelphia, PA 19104 USA; 4 Department of Medicine, Perelman School of Medicine at the University of Pennsylvania, Philadelphia, PA 19104 USA

**Keywords:** Quiescence, Dormancy, Residual disease, Recurrence, Gene expression, Breast cancer, Targeted therapy, EMT, Stem cells

## Abstract

**Background:**

Breast cancer mortality is principally due to tumor recurrence, which can occur following extended periods of clinical remission that may last decades. While clinical latency has been postulated to reflect the ability of residual tumor cells to persist in a dormant state, this hypothesis remains unproven since little is known about the biology of these cells. Consequently, defining the properties of residual tumor cells is an essential goal with important clinical implications for preventing recurrence and improving cancer outcomes.

**Methods:**

To identify conserved features of residual tumor cells, we modeled minimal residual disease using inducible transgenic mouse models for HER2/neu and Wnt1-driven tumorigenesis that recapitulate cardinal features of human breast cancer progression, as well as human breast cancer cell xenografts subjected to targeted therapy. Fluorescence-activated cell sorting was used to isolate tumor cells from primary tumors, residual lesions following oncogene blockade, and recurrent tumors to analyze gene expression signatures and evaluate tumor-initiating cell properties.

**Results:**

We demonstrate that residual tumor cells surviving oncogenic pathway inhibition at both local and distant sites exist in a state of cellular dormancy, despite adequate vascularization and the absence of adaptive immunity, and retain the ability to re-enter the cell cycle and give rise to recurrent tumors after extended latency periods. Compared to primary or recurrent tumor cells, dormant residual tumor cells possess unique features that are conserved across mouse models for human breast cancer driven by different oncogenes, and express a gene signature that is strongly associated with recurrence-free survival in breast cancer patients and similar to that of tumor cells in which dormancy is induced by the microenvironment. Although residual tumor cells in both the *HER2/neu* and *Wnt1* models are enriched for phenotypic features associated with tumor-initiating cells, limiting dilution experiments revealed that residual tumor cells are not enriched for cells capable of giving rise to primary tumors, but are enriched for cells capable of giving rise to recurrent tumors, suggesting that tumor-initiating populations underlying primary tumorigenesis may be distinct from those that give rise to recurrence following therapy.

**Conclusions:**

Residual cancer cells surviving targeted therapy reside in a well-vascularized, desmoplastic microenvironment at both local and distant sites. These cells exist in a state of cellular dormancy that bears little resemblance to primary or recurrent tumor cells, but shares similarities with cells in which dormancy is induced by microenvironmental cues. Our observations suggest that dormancy may be a conserved response to targeted therapy independent of the oncogenic pathway inhibited or properties of the primary tumor, that the mechanisms underlying dormancy at local and distant sites may be related, and that the dormant state represents a potential therapeutic target for preventing cancer recurrence.

**Supplementary Information:**

The online version contains supplementary material available at 10.1186/s13058-021-01416-9.

## Background

The vast majority of tumor cells in early-stage breast cancer patients are eliminated by the combination of surgery, radiation therapy and adjuvant therapies. Despite these treatments, many patients harbor residual tumor cells—termed minimal residual disease (MRD)—at local or distant sites that can survive therapy and persist in a latent state for many years. These cells constitute the reservoir from which recurrent cancers arise. Importantly, while primary breast cancers can be cured, recurrent breast cancers cannot. Consequently, therapeutic targeting of MRD could represent a tractable approach to preventing breast cancer recurrence and its associated mortality by depleting, or otherwise inhibiting, the reservoir of residual tumor cells that persists following therapy.

Consistent with a precursor-product relationship between MRD and recurrent tumors, the presence of disseminated tumor cells (DTCs) in bone marrow following treatment is an independent prognostic factor for recurrence-free survival in multiple cancer types, including breast cancer, wherein bone marrow DTCs are associated with a higher risk of loco-regional and distant recurrence, and poorer breast cancer-specific survival and overall survival [1–4]. Since many breast cancer patients pass through a protracted latency period prior to recurrence, MRD has been hypothesized to exist in a dormant state, which could reflect either tumor mass dormancy or cellular dormancy [5–9]. Tumor mass dormancy occurs when a population of cancer cells exhibits balanced rates of proliferation and cell death due to immune surveillance or lack of an adequate blood supply, such that macroscopic tumors fail to form. In contrast, cellular dormancy occurs when tumor cells reversibly exit the cell cycle, but remain capable of re-initiating tumor growth.

Certain clinical observations support the idea that breast cancers may pass through a stage of cellular dormancy prior to recurrence that, if true, would suggest a therapeutic window through which to target MRD and thereby prevent tumor recurrence [5, 6, 9–14]. If, however, tumor relapses arise not from quiescent tumor cells but from proliferative subclinical micrometastases that fail to expand due to immune surveillance or lack of an adequate vasculature, cellular dormancy might be less likely to constitute a tractable therapeutic target.

In light of this uncertainty, determining whether cellular dormancy plays a role in tumor recurrence could shed light on whether MRD represents a stage of neoplastic progression that might be susceptible to therapies targeting this quiescent state. Achieving this goal, however, has been challenging since the process by which residual tumor cells give rise to recurrent breast cancers cannot readily be studied in patients due to the limited accessibility of residual tumor cells, reliance on the use of epithelial cell surface markers to identify them, and the inability to functionally distinguish cellular dormancy from irreversible cell cycle arrest in patients.

Given these challenges, increasing efforts have focused on animal models that faithfully recapitulate essential aspects of breast cancer dormancy and recurrence. To that end, we have developed a series of conditional genetically engineered mouse (GEM) models for breast cancer progression that permit the inducible expression of clinically relevant oncogenes in the mammary epithelium of transgenic mice treated with doxycycline [15–18]. Transgenic mice develop invasive mammary adenocarcinomas that spontaneously metastasize to the lungs and other sites. In addition, primary tumors regress to a non-palpable state following oncogene pathway downregulation [15, 17, 19] as a consequence of oncogene addiction, as is also observed in breast cancer patients treated with targeted therapies [20, 21]. Also similar to patients, tumor regression in mice leaves behind a small population of residual cancer cells that can spontaneously give rise to recurrent tumors [16–18, 22].

Recently, we have used these models to identify pathways that functionally contribute to mammary tumor recurrence in mice [18, 23–25]. Consistent with their effects on tumor recurrence in mice, interrogation of gene expression data sets representing > 4000 human breast cancers revealed that elevated Notch activity, SPSB1/c-MET activity, and Snail expression—as well as decreased Par-4 expression—are associated with an increased risk of distant relapse in patients. In addition, obesity in this GEM model was associated with accelerated rates of recurrence, as is also observed in patients [26]. These findings in mice, coupled with clinical observations that local recurrence is strongly associated with an increased risk of distant relapse and mortality in breast cancer patients [27–30] and that the timing of local and distant tumor relapse following surgery are similar [31, 32], suggest that the mechanisms by which residual tumor cells survive and recur may be conserved irrespective of whether tumor cells persist locally or at distant sites. These and other findings indicate that GEM models for local recurrence may be informative for mechanisms underlying distant, as well as local, relapse in patients.

In light of the clinical relevance of these GEM models for tumor recurrence, we now define the biological properties of the population of residual tumor cells that gives rise to recurrent tumors. Specifically, in *HER2/neu* or *Wnt1* tumor models we find that lineage-marked residual tumor cells surviving oncogenic pathway inhibition exhibit cellular dormancy, retain the ability to re-enter the cell cycle after extended periods of quiescence, and express markers shared with mammary stem cells as well as tumor-initiating cells. Further supporting the clinical relevance of dormancy in these models, a gene expression signature derived from FACS-purified dormant residual tumor cells in mice is strongly associated with the risk of late relapse in breast cancer patients. Our studies additionally demonstrate that cellular dormancy induced by targeted therapy occurs in human breast cancer xenografts treated with anti-HER2 and anti-ER therapies, and at metastatic sites in tumor-bearing mice. In aggregate, our observations indicate that cellular dormancy may be a conserved mechanism allowing residual tumor cells to escape oncogenic pathway inhibition and provide new insights into biological properties of MRD relevant to tumor recurrence.

## Methods

### Mice

Animal care and experiments were performed with the approval of, and in accordance with, guidelines of the University of Pennsylvania IACUC. Primary and recurrent tumors in transgenic and *nu/nu* mice were generated, and recurrence assays were performed, as described [17, 18].

Xenograft studies were performed in *NOD/scid/Il2γnull* (*NSG*; Jackson Laboratory, stock #005557) mice that had been oophorectomized and implanted subcutaneously with 17β-estradiol pellets. For HER2 blockade, *NSG* mice were administered 100 mg/kg lapatinib by oral gavage 5 days/week, and 20 mg/kg trastuzumab and 20 mg/kg pertuzumab three times per week. ER signaling was inhibited by removal of 17β-estradiol pellets. NSG mice were sacrificed 72 h after treated tumors regressed to a non-palpable state.

To generate syngeneic primary tumors and residual lesions, tumor cells from independently arising primary tumors were injected into *nu/nu* mice on doxycycline to generate orthotopic primary tumors. Doxycycline was then withdrawn from a subset of tumor-bearing mice to generate syngeneic orthotopic residual lesions derived from the same donor tumor.

### Tissue culture and reagents

Primary tumor cells were cultured and stable H2B-eGFP expression achieved as described [18]. BT474-M1 tumor cells were a gift from Dr. Mien-Chie Hung. Cells were grown in media consisting of 10% fetal bovine serum (FBS, Gibco) and 1% Penicillin/Streptomycin in Dulbecco’s modified Eagle’s medium (DMEM)/F12 (GIBCO 11330-032). Stable GFP expression was achieved by transduction with GFP Bsd Lentiviral particles (Gentarget, LVP001) followed by culture in media containing 10 μg/ml blasticidin.

### Immunofluorescence and direct fluorescence

Mammary glands bearing minimal residual lesions (MRLs) were harvested, embedded in optimal cutting temperature (OCT), and frozen. Then, 8-μm tissue sections were either fixed and permeabilized in dehydrated acetone at − 20 °C for 10 min, or fixed in 4% paraformaldehyde for 10 min at room temperature (RT) and permeabilized in 0.1% Triton-X-100 in phosphate-buffered saline (PBS) for 20 min. Following fixation and permeabilization, sections were washed 2 × 5 min in PBS, blocked for 1 h with 10% normal goat serum with 3% BSA in PBS and incubated overnight (O/N) at 4 °C with primary antibody diluted in blocking buffer.

Primary antibodies used to stain tissues fixed in paraformaldehyde were as follows: CD31 (1:200, AB28364, AbCam); CK5 (1:1000, PRB-160P, Covance); CK14 (1:10,000, PRB-155P, Covance); CK19 (1:50, AB15463, AbCam); Pan-CK Ab (1:100, SC-15367, Santa Cruz); E-Cadherin (1:100, 13–1900, Zymed); P-Cadherin (1:50, 13–200, Zymed); p63 (1:500, AB53039, AbCam); Collagen Type-I (1:250, AB34710, AbCam); Ki67 (1:50, M7249, Dako); and BrdU (1:200, OBT0030, AbD Serotec). Primary antibodies used to stain tissues fixed in acetone were as follows: EpCAM (1:100, 14–5791, eBioscience) and CK18 (1:100, NB110–56910, Novus Biologicals).

For BrdU staining, following fixation and permeabilization, sections were incubated with 2N HCl for 10 min at 25 °C and then 20 min at 37 °C, followed by incubation in 0.1 M sodium tetraborate for 15 min, and then blocked, as above, and stained with primary antibody. For staining of tissues with primary antibodies raised in mice, specifically those against Fibronectin (1:200, 610,077, BD Transduction), the M.O.M.™ Immunodetection Kit (Vector Laboratories) was used with Avidin/Biotin Blocking Kit (Vector Laboratories). Following O/N incubation with primary antibody, tissues were washed 2 × 10 min in PBS, and incubated for 1 h at RT with AlexaFluor-conjugated secondary antibody (1:1000 dilution, Life Technologies Corporation). Sections were then washed 10 min in PBS, 10 min in PBS with a 1:10,000 dilution of Hoechst 33258 (Sigma-Aldrich Co.), and 5 min in PBS. Sections were mounted in ProLong® Gold Antifade Reagent (Molecular Probes®, Life Technologies Corporation), sealed with clear nail polish, and allowed to dry.

Slides were imaged with a Leica TCS SP5 (Leica Microsystems) confocal microscope using Leica Application Suite Advanced Fluorescence (LAS AF, Leica Microsystems) software. Images in Fig. 3 showing Ki67 or BrdU staining of H2B-eGFP-labeled *HER2/neu-Prim1* tumor cells are presented as a masked image, wherein a binary mask is generated from the GFP channel and applied to the Ki67 or BrdU channel, so that only Ki67 or BrdU co-staining with tumor cells is shown. To avoid selection bias that can be introduced during analysis of photomicrographs, images used for quantification represented either the entire MRL, or 1 mm × 1 mm of the primary tumor, generated as a mosaic of multiple smaller fields of view.

### Intravital labeling

Pimonidazole HCl (Hypoxyprobe™-1, NPI Inc.) was resuspended in PBS at 20 μg/μl and injected i.v. at 60 mg/kg 90 min prior to sacrifice. Hoechst 33342 (230001000, Thermo Fisher Scientific Inc.) was resuspended in PBS at 5 μg/μl and injected i.v. at 20 mg/kg 15 min prior to sacrifice. Lectin-AF647 was prepared by resuspending 1 mg *Lycopersicon esculentum* Lectin (B-1175, Vector Labs, Inc) in 425 μl of PBS and mixing with 75 μl of Streptavidin, AlexaFluor® 647 Conjugate (S-32357, Life Technologies Corporation). Lectin-AF647 was centrifuged briefly to remove protein aggregates and was injected i.v. 10 min prior to sacrifice. Mammary glands bearing MRLs 28d following oncogene deinduction were harvested, along with livers, at sacrifice and placed directly into optimal cutting temperature (OCT, Tissue-Tek, VWR). Slides were imaged with a Leica microscope and software, as above.

### Image quantification

Images were quantified using CellProfiler (Broad Institute) software. Briefly, to quantify Ki67 or BrdU, a mask was generated, as above, using H2B-eGFP nuclei from a mosaic image. The number of nuclei that stained positive for Ki67 or BrdU was determined using this mask. The fraction of tumor cells expressing BrdU or Ki67 was calculated as the total number of nuclei that stained positive for Ki67 or BrdU divided by the total number of nuclei.

### Flow cytometry and fluorescence-activated cell sorting (FACS)

Tissues harvested from mice were digested enzymatically with collagenase/hyaluronidase (Stemcell Technologies) for 90 min at 37 °C, followed by vigorous pipetting in DNAse (Worthington Biochemical Corporation) and Dispase (Stemcell Technologies) for 5 min at 37 °C. Tissue digests were strained through a 40-μm filter (BD Falcon). Single-cell suspensions were stained with fluorophore-conjugated antibodies for 30 min at 4 °C in 1% bovine serum albumin (BSA, Sigma-Aldrich Co.) and 5 mM ethylenediaminetetraacetic acid disodium salt (EDTA; Sigma-Aldrich Co.) in PBS. Following staining, cells were washed twice in FACS diluent (PBS with 1% BSA) and 5 mM EDTA and resuspended in FACS diluent with 1 μg/ml DAPI (Sigma-Aldrich Co.) for identifying viable cells.

Flow cytometry was performed on a BD FACSCanto™ system using BD FACSDiva™ software (BD Biosciences). Compensation was performed using AbC™ anti-mouse bead kit (A10344, Life Technologies Corporation) and anti-rat/hamster bead kit (A10389, Life Technologies Corporation), according to the manufacturer’s instructions. All steps for flow cytometry, FACS, and limiting dilution experiments were performed at 4 °C, with the exception of the enzymatic digestion, which was performed at 37 °C.

Fluorophore-conjugated antibodies used were as follows: CD45-AF700 (1:50, 560510, BD Biosciences); CD49e-PE (1:50, 557447, BD Pharmingen); CD24-Pe-Cy7 (1:200, 560536, BD Biosciences); EpCAM-APC-Cy7 (1:50, 118218, BioLegend); and PDGFR-β-APC (1:50, 17-1402, eBiosciences).

Data were analyzed using FlowJo. Dead cells, debris, and doublets were removed by sequentially analyzing gates for DAPI, SSC-A vs. FSC-A, SSC-W vs. SSC-H, and FSC-W vs. FSC-H staining, respectively. For tumors harvested from mice bearing a YFP-reporter, tumor cells were defined as YFP+CD45−DAPI− cells, excluding all dead cells, debris, and doublets as above.

### Human breast cancer microarray data sets

Publicly available microarray data for 4463 patients contained within 17 human primary breast cancer data sets [33–48] were downloaded from NCBI GEO or original authors’ websites along with the corresponding clinical annotations. Microarray data were converted to base 2 log scale where necessary. Affymetrix microarray data were re-normalized using Robust Multi-array Average (RMA [49]) when .CEL files were available.

### Gene expression analysis of purified tumor and stromal/immune cells

Tissue digestion was performed as above for flow cytometry with the exception that digestion in collagenase/hyaluronidase was limited to 20 min. To minimize transcriptional changes, tissue/cells were maintained on ice or at 4 °C for all other steps prior to resuspension in Trizol after sorting, which was based solely on GFP expression and DAPI staining.

Removal of dead cells, debris, and doublets was performed as above for flow cytometry. Cell sorting was performed on a BD FACSVantage cell sorter. Cells were sorted into complete media (including doxycycline for primary tumor cells), chilled to 4 °C, and maintained on ice. Immediately following sorting, cells were pelleted and resuspended in TRIZOL. Phenol/chloroform extraction was used in conjunction with the QIAGEN RNeasy kit to isolate mRNA. Isolated RNA was analyzed on an Agilent Bioanalyzer, and only samples with an RNA integrity number of at least 7.0 were selected for subsequent amplification and expression analysis. In total, 500 pg of RNA was used as the input for whole transcriptome amplification (WTA) with NuGEN Ovation Pico WTA V2.0 kits.

An expression heat map of ribosomal protein gene expression was generated using GENE-E (www.broadinstitute.org/cancer/software/GENE-E/).

### Gene expression signatures

A dormancy gene expression signature was generated from microarray data sets of *HER2/neu* and *Wnt1* tumor murine mammary tumor residual lesion models. The signature consists of genes differentially expressed (|fold-change| > 1.5, FDR < 0.1) between residual lesions and primary tumors and between residual lesions and recurrent tumors in a concordant manner across both models. Differential expression analyses were performed using Cyber-T [50]. Signature scores were calculated in each of the human breast cancer microarray data sets using the relative expression of the signature genes and a scoring method described previously [51].

To estimate relative proliferation levels in human breast cancer samples, we generated a gene expression signature containing 224 genes from the overlap of two gene sets: (1) 651 cell cycle-regulated genes in HeLa cells [52] and (2) 1882 serum responding genes in human fibroblasts identified using data in [53]. The serum responding genes were identified by differential expression analysis between the 0.1% and the 10% serum groups using Cyber-T [50] at a false discovery rate of 10%. In each human breast cancer data set, levels of proliferation were estimated using the 224 genes and a scoring method described previously [51], in which each gene was weighted using its log fold-change between the 0.1% and the 10% serum groups in [53].

As comparisons to the association of this dormancy signature with recurrence-free survival, signature scores in the human breast cancer microarray data sets were also calculated using nine publicly available gene expression signatures related to breast cancer prognosis or tumor dormancy, including Oncotype DX [54], the Molecular Grade Index [55], the 70-gene prognostic signature [56], the wound-response signature [53], a p38-related dormancy signature [57], a two-gene prognostic signature [58], a HR-neg/Triple-negative breast cancer signature [59], an immune response signature [60], and a 51-gene prognostic signature [61]. With exception of the 70-gene signature, signature scores were calculated as weighted averages on the *z*-scores of the signature genes’ expression data, in which the weights are the direction of association (1 or − 1) for each gene with the signature. The signature scores for the 70-gene signature were generated using a publicly available implementation (in the “genefu” R package) of the methodology used in the original publication.

### Gene expression signature scores and recurrence-free survival

Within each data set, the effect size of the association between gene expression signatures and 5-year relapse-free survival was estimated using the hazard ratio from Cox proportional hazards regression, in which the signature scores were modeled as a binary variable by dichotomizing the samples in each data set by the median of the signature scores. Effect size estimates were combined across data sets by meta-analysis using the inverse-variance weighting method [62]. Between-study homogeneity of survival association was tested using chi-squared test on Cochran’s Q statistic [63], for which a *p* value of less than 0.05 was interpreted as evidence of significant heterogeneity. In the presence of significant heterogeneity, the random-effect model [64] was used for meta-analysis. In the absence of significant heterogeneity, the fixed-effect model [65] was used. Effect size estimates from multivariate models in which the proliferation signature scores were included as a covariate were similarly summarized by meta-analysis. For data sets in which relapse-free survival information was not available, distant metastasis-free survival or disease-specific survival information, when available, was used for survival analysis.

The association between signature scores and late (> 5 years) relapse-free survival was analyzed in a similar fashion using the approach describe above for 5-year relapse-free survival, except that only patients who remained relapse-free in their first 5 years were included in the analysis of late relapse.

### Tumor-initiating cell assays

Isolated viable tumor cells were injected into the mammary fat pads of *nu/nu* mice, and mice were monitored for 4 months for tumor development. Tumor-initiating cell (TIC) frequency was calculated based on the fraction of mice that developed tumors when injected with a given number of tumor cells [66].

## Results

### The kinetics of tumor recurrence following oncogene inhibition suggest dormant residual disease

We previously described conditional bitransgenic mouse models for HER2/neu and Wnt1-induced mammary tumorigenesis that recapitulate key features of breast cancer progression as it occurs in patients [16–18, 22]. Doxycycline administration to *MMTV-rtTA;TetO-HER2/neu* (*MTB;TetO-HER2/neu*) and *MMTV-rtTA;TetO-Wnt1* (*MTB;TetO-Wnt1*) mice results in formation of primary mammary adenocarcinomas and, analogous to the treatment of human cancers with targeted therapies, oncogenic pathway inhibition in tumor-bearing mice results in primary tumor regression due to oncogene addiction [17, 19]. However, mirroring the occurrence of resistance and relapse in patients treated with targeted therapies, mice develop spontaneous recurrent tumors with stochastic kinetics following a variable period of latency (Additional file 1: Fig. S1a, c) [17, 18]. As such, these models provide an opportunity to investigate the biological properties of residual tumor cells that survive targeted therapy and give rise to tumor recurrence.

To determine whether adaptive immunity is required for sustained regression of mammary adenocarcinomas, we injected limited passage tumor cells derived from primary mammary tumors arising in *MTB;TetO-HER2/neu (HER2/neu-Prim1)* or *MTB;TetO-Wnt1* (*Wnt1-Prim1*) mice into the mammary fat pads of immunocompromised female *nu/nu* mice maintained on doxycycline. Following primary tumor formation, doxycycline was withdrawn to induce oncogene downregulation and tumor regression. Paralleling our findings in intact mice, orthotopic tumors recurred with stochastic kinetics following a variable latency period (Additional file 1: Fig. S1b, d), indicating that a competent adaptive immune system is not required for sustained periods of tumor regression following oncogenic pathway inhibition.

Notably, in both orthotopic and intact mouse models, tumors recurring after either short or long intervals exhibited similar growth rates (Additional file 1: Fig. S1e) [18]. This pattern is reminiscent of observations in breast cancer patients and suggests the potential existence of a dormant phase prior to tumor recurrence [5].

To address whether mice bearing fully regressed mammary tumors harbor dormant residual disease, we re-administered doxycycline to intact *MTB;TetO-HER2/neu* mice whose primary tumors had regressed to a non-palpable state following doxycycline withdrawal, but had not exhibited spontaneous tumor recurrences when maintained off doxycycline for a period of 6 months. In contrast to the 17-week latency for spontaneous tumor recurrence, doxycycline-induced re-expression of the *HER2/neu* transgene resulted in recurrent tumor formation at the original sites of primary tumors within 2 weeks in all mice (Additional file 1: Fig. S1f). This finding demonstrates that mice bearing tumors that have regressed to a non-palpable state harbor residual cancer cells capable of giving rise to recurrent tumors, and suggests that these cells exist in a latent state.

### Identifying residual tumor cells in residual lesions

**Fig. 1:**
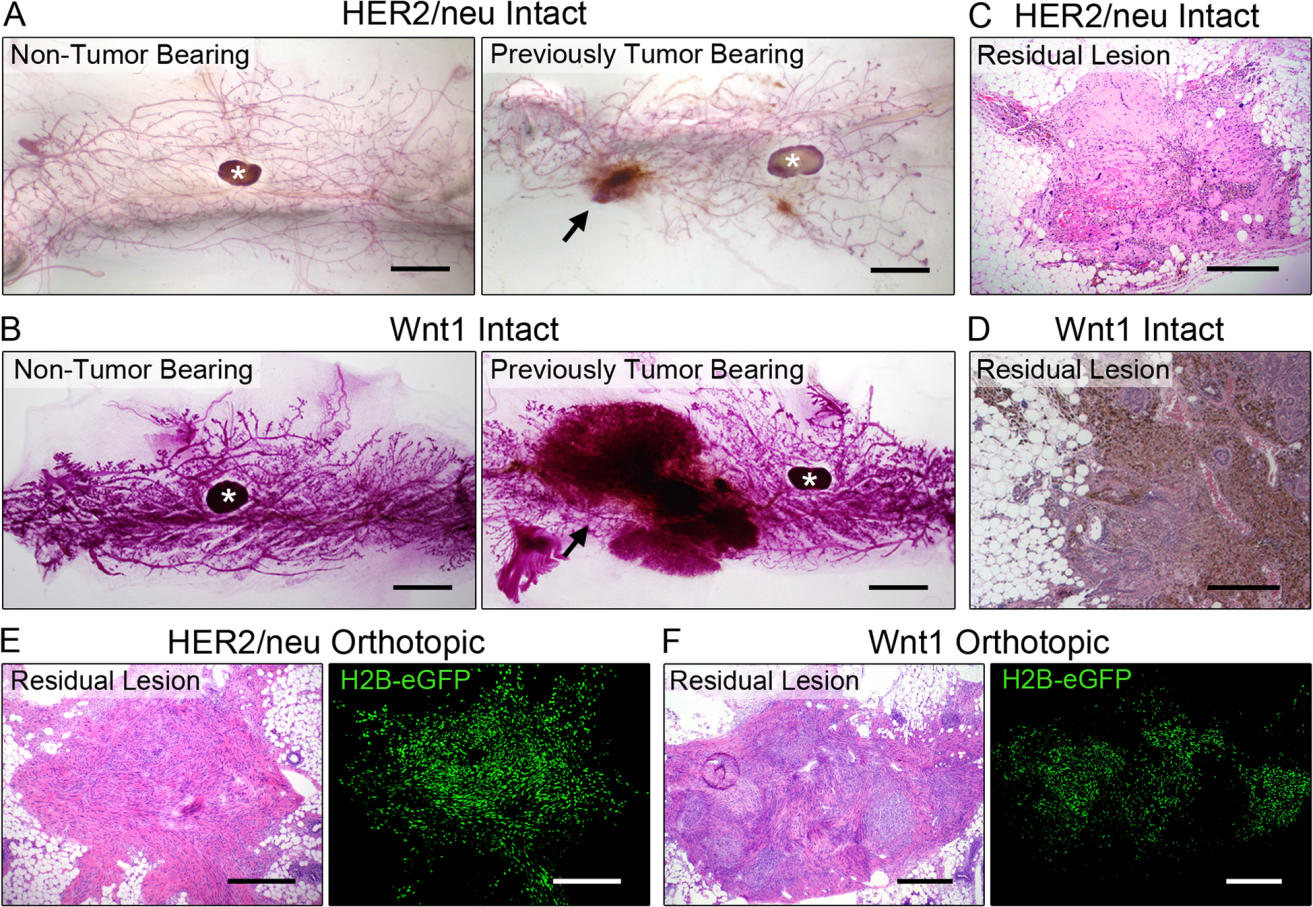
Identification of residual tumor cells following oncogenic pathway inhibition. **a**, **b** Carmine staining of mammary glands following oncogenic pathway inhibition in **a** *MTB;TetO-HER2/neu* and **b** *MTB;TetO-Wnt1* models. (*) lymph node. **c**, **d** H&E-stained sections of intact **c** *MTB;TetO-HER2/neu* and **d** *MTB;TetO-Wnt1* MRLs. **e**, **f** H&E-stained sections (left) and fluorescence microscopy of H2B-eGFP-labeled tumor cells (right) in orthotopic **e** *HER2/neu-Prim1* and **f** *Wnt1-Prim1* MRLs. Scale bars (**a**, **b**) 750 μm and (**c**–**f**) 250 μm

To identify residual tumor cells, previously tumor-bearing *MTB;TetO-HER2/neu* and *MTB;TetO-Wnt1* mice were sacrificed 56 days following doxycycline withdrawal. Examination of carmine-stained mammary glands revealed the presence of histopathological lesions in mammary glands that had previously harbored primary tumors, but not in non-tumor-bearing mammary glands (Fig. 1a, b, arrows). Hematoxylin and eosin (H&E) staining of previously tumor-bearing glands revealed that residual foci were densely cellular (Fig. 1c, d).

To facilitate analysis of these cells, H2B-eGFP-expressing *HER2/neu-Prim1* or *Wnt1-Prim1* cells were used to generate orthotopic residual disease. H&E staining of mammary glands bearing residual lesions revealed cellular foci within a dense eosinophilic extracellular matrix (ECM) similar to that identified in intact mice (Fig. 1e). Consistent with this, immunofluorescence staining (IF) for fibronectin and type I collagen revealed an abundant desmoplastic stroma within residual foci (Additional file 2: Fig. S2). Fluorescence microscopy confirmed the presence of eGFP-labeled tumor cells within these residual foci, which we term minimal residual lesions (MRLs) (Fig. 1f).

### Residual tumor cells exhibit cellular dormancy

Based on the stochastic kinetics of tumor recurrence and similar growth rates of recurrent tumors irrespective of their latency to recurrence, we hypothesized that residual tumor cells surviving oncogene downregulation exist in a state of cellular dormancy. To test this hypothesis, we sacrificed mice bearing orthotopic H2B-eGFP-labeled *HER2/neu-Prim1* primary tumors or MRLs at either 28 days or 56 days following doxycycline withdrawal.

**Fig. 2:**
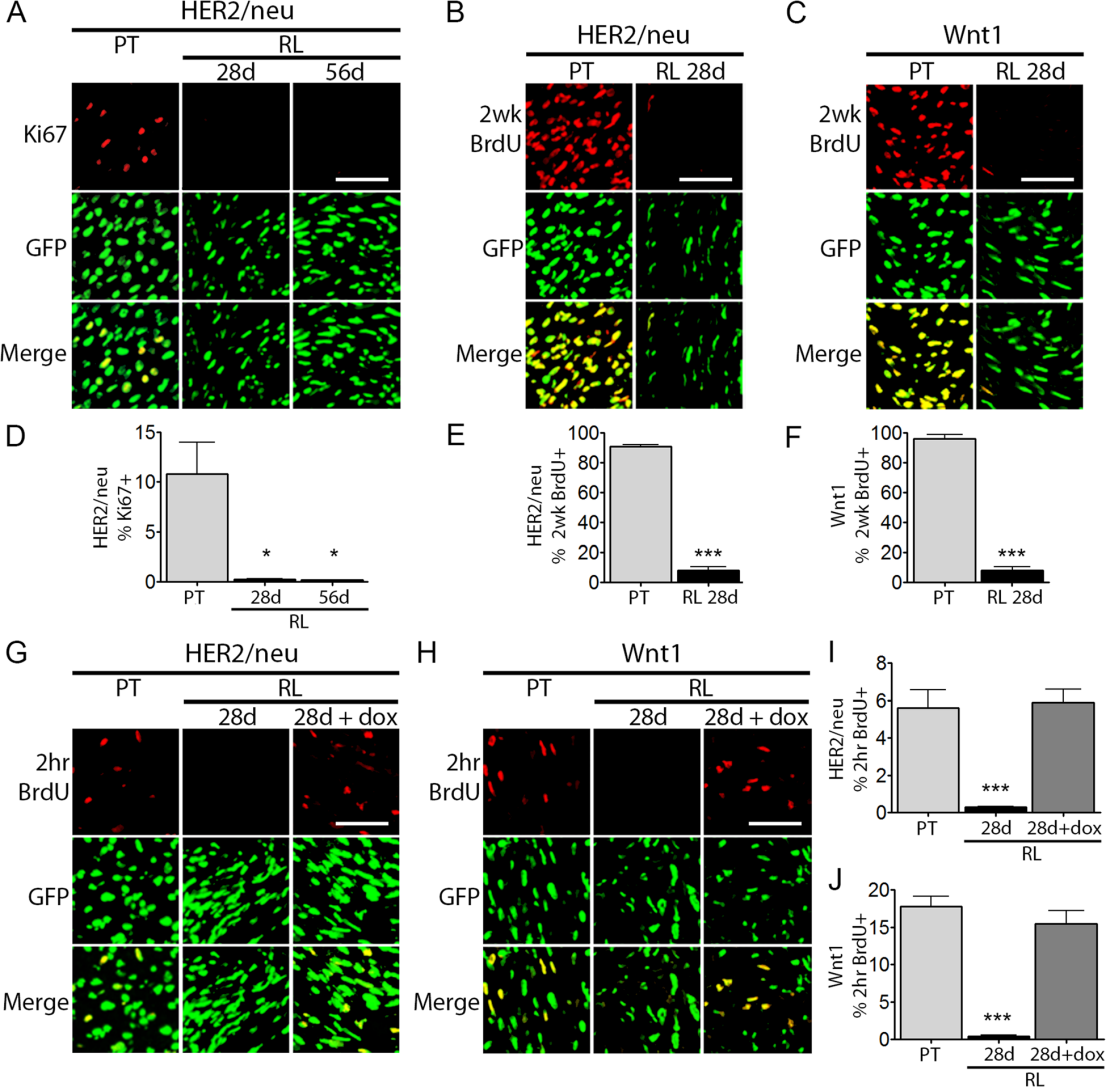
Residual tumor cells are quiescent. **a** Immunofluorescence (IF) staining for Ki67 on sections from H2B-eGFP-labeled orthotopic *HER2/neu-Prim1* primary tumors (PT, left) and residual lesions (RL) 28 days (middle) or 56 days (right) after HER2/neu deinduction. **b**, **c** IF for BrdU on sections from H2B-eGFP-labeled *HER2/neu-Prim1* (**b**) or *Wnt1-Prim1* (**c**) orthotopic primary tumors (left) or MRLs 28 days after oncogene deinduction (right) in mice labeled with BrdU for 2 weeks prior to sacrifice. **d** Quantification of H2B-eGFP-labeled tumor cells staining positive for Ki67 in **a**. **e**, **f** Quantification of H2B-eGFP-labeled tumor cells staining positive for BrdU in (**b**, **c**), respectively. **g**, **h** IF staining for BrdU on sections from H2B-eGFP-labeled orthotopic primary tumors (left), or MRLs 28 days after deinduction (middle) or 72 h following reinduction (right). Mice were labeled with BrdU for 2 h prior to sacrifice. **i**, **j** Quantification of H2B-eGFP-labeled tumor cells staining positive for BrdU in (**g**, **h**), respectively. Scale bars (**a**-**c**, **g**, **h**) 50 μm. *p value vs. primary tumor (PT) < 0.05. ***p value vs. primary tumor (PT) < 0.001

Evaluation of Ki67 staining revealed that residual tumor cells in MRLs at either 28 days or 56 days following HER2/neu downregulation exhibited rates of Ki67 positivity more than 40-fold lower than primary tumor cells (Fig. 2a, d). This finding indicates that residual tumor cells are quiescent following oncogene downregulation.

To distinguish slow-cycling cells from tumor cells undergoing prolonged cell cycle arrest, as would be anticipated for dormant tumor cells, we evaluated tumor cell proliferation rates over a 2-week interval. Orthotopic primary tumors and MRLs were generated, as above, from H2B-eGFP-labeled *HER2/neu-Prim1* or *Wnt1-Prim1* tumor cells, and osmotic pumps were employed to deliver bromodeoxyuridine (BrdU) for 2 weeks prior to sacrifice.

Whereas greater than 90% of *HER2/neu-Prim1* and *Wnt1-Prim1* primary tumor cells were labeled with BrdU over the 2-week period prior to sacrifice, fewer than 9% of *HER2/neu-Prim1* and *Wnt1-Prim1* residual tumor cells incorporated BrdU over the same time period (Fig. 2b, c, e, f). These findings indicate that the majority of residual tumor cells from either HER2/neu or Wnt1-induced mammary tumors reside in a latent G_0_-like state following oncogene downregulation, or are extremely slow cycling.

We next wished to confirm that residual tumor cells are dormant—existing in a reversible state of cellular quiescence—by demonstrating that these cells have not irreversibly exited the cell cycle, as might occur if cells had undergone senescence or terminal differentiation. To address this, we re-administered doxycycline for 72 h to mice bearing 28-day MRLs derived from H2B-eGFP-labeled *HER2/neu-Prim1* or *Wnt1-Prim1* tumors.

In both the *HER2/neu* and *Wnt1*-driven models, residual tumor cells in which oncogene expression had been reactivated proliferated at rates similar to primary tumor cells, as measured by 2 h BrdU incorporation. In contrast, residual tumor cells in which oncogene expression had not been reactivated proliferated at ~ 20-fold lower rates (Fig. 2g–j). Together, these findings demonstrate that residual tumor cells exist in a reversible state of cellular quiescence, referred to as cellular dormancy, following oncogenic pathway inhibition.

### Residual lesions are well-vascularized

**Fig. 3:**
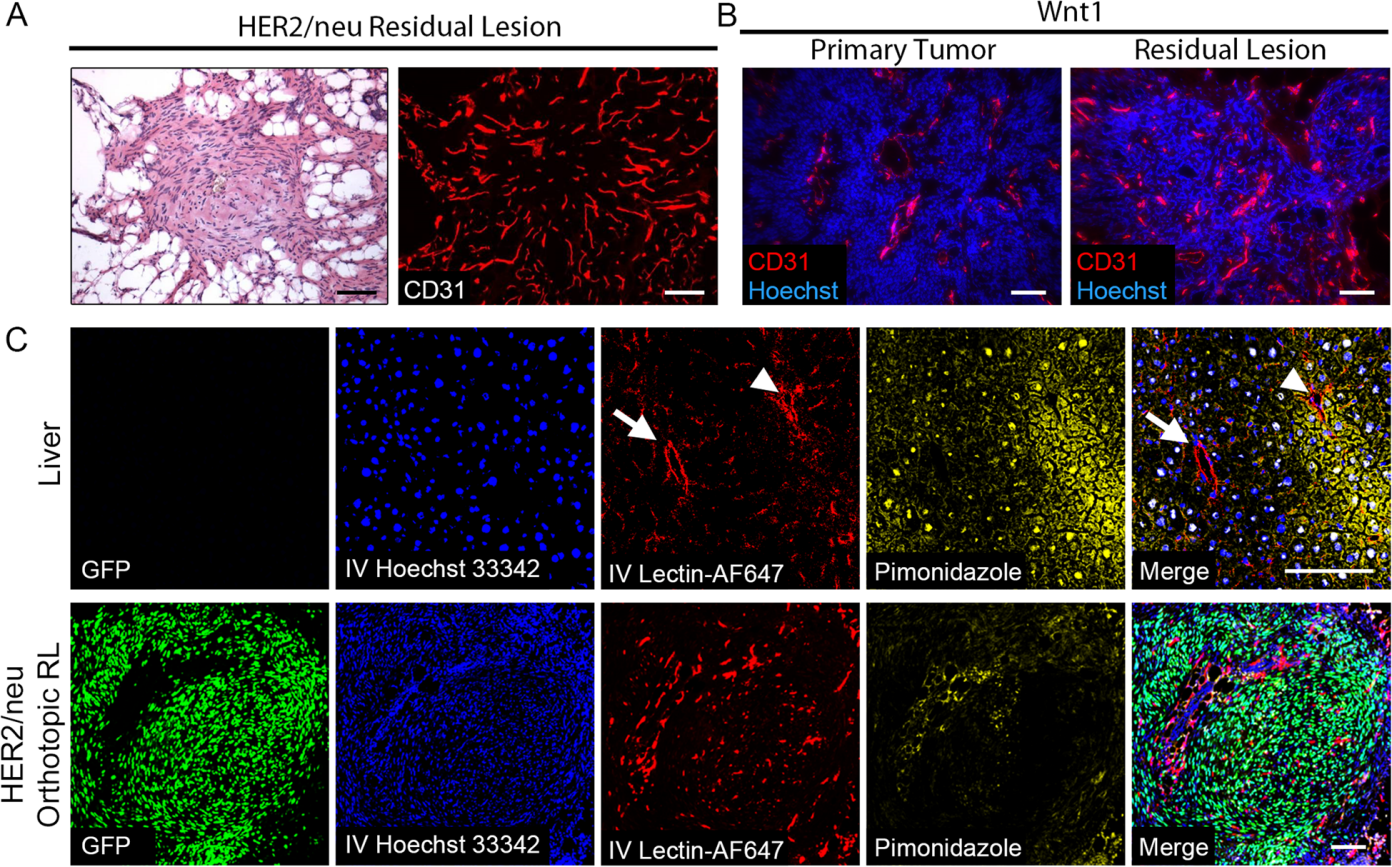
Minimal residual lesions are well-vascularized and not hypoxic. **a** H&E (left) and IF staining for CD31 (right) on sections of a representative *HER2/neu-Prim1* orthotopic MRL. **b** IF staining for CD31 on sections of an intact *MTB;TetO-Wnt1* primary tumor (left) or MRL (right). **c** Fluorescence microscopy of liver (top) or orthotopic MRL (bottom) from same mouse for H2B-eGFP, i.v. injected Hoechst 33342 and Lectin-AF647, and immunofluorescence staining for pimonidazole. Arrowhead denotes positive pimonidazole staining surrounding the hepatic vein; arrow denotes absence of pimonidazole staining surrounding the hepatic artery. Scale bars: 100 μm for all images shown

Prior reports have suggested that sustained tumor regression following oncogenic pathway inhibition may be attributable to angiogenic dormancy, a type of tumor mass dormancy driven by a lack of functional vascularization [67]. In contrast, immunofluorescence (IF) staining for the endothelial cell marker CD31 demonstrated that both *HER2/neu* and *Wnt1* residual lesions are densely vascularized (Fig. 3a, b), with blood vessel densities similar to, or greater than, those present within actively growing primary tumors (Fig. 3b). Moreover, virtually all CD31 staining co-localized with Lectin-Streptavidin-AlexaFluor647 (Lectin-AF647) that had been injected intravenously (i.v.) to label patent blood vessels (Additional file 3: Fig. S3), demonstrating that blood vessels within MRLs are well perfused.

To extend these findings, mice bearing orthotopic MRLs were injected i.v. with Lectin-AF647, Hoechst 33342, and pimonidazole prior to sacrifice. In contrast to low-oxygen tension regions of the liver adjacent to hepatic veins (Fig. 3c, arrowhead), and similar to well-oxygenated regions adjacent to the hepatic arterial vasculature (Fig. 3c, arrow), virtually no pimonidazole staining was observed in MRLs, indicating that residual tumor cells within the MRL do not exist within a hypoxic microenvironment (Fig. 3c). Furthermore, i.v. injected Hoechst 33342 efficiently labeled the nuclei of cells in both liver and MRLs, demonstrating that small molecules within the circulation can diffuse out of the vasculature and efficiently gain access to residual tumor cells within MRLs (Fig. 3c).

Together, our findings in both *HER2/neu* and *Wnt1* models that tumor cells surviving oncogenic pathway downregulation are quiescent, despite a richly vascularized, non-hypoxic microenvironment, suggest that these cells exist in a state of cellular dormancy. This, in turn, suggests that residual tumor cell dormancy observed following oncogenic pathway inhibition is not due to angiogenic insufficiency or inadequate nutrient delivery.

### Dormant residual tumor cells exhibit a conserved gene expression profile implicating the extracellular matrix and mTOR, uPAR, TGFβ, and thrombospondin pathways

**Fig. 4:**
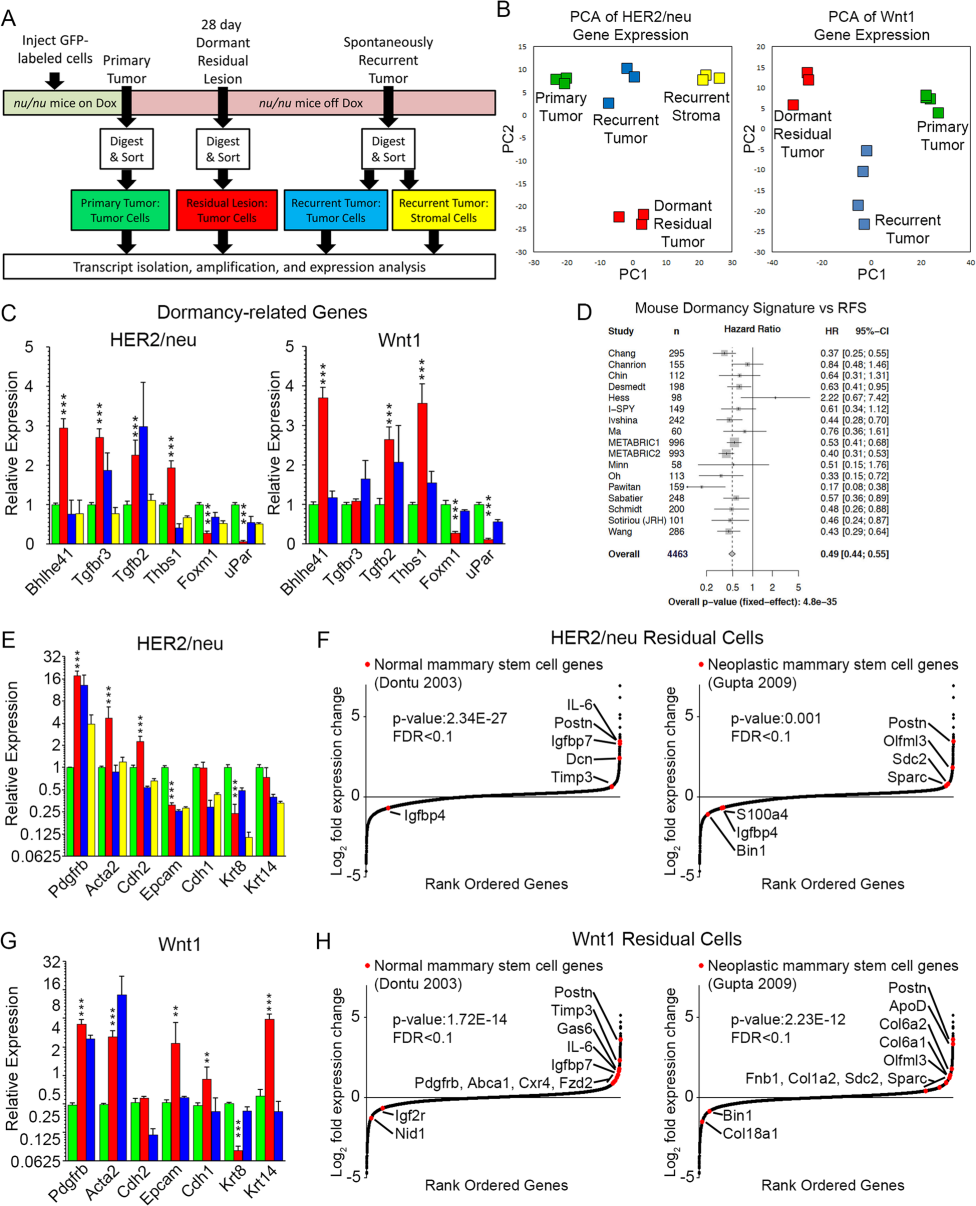
Residual tumor cells express genes associated with EMT and mammary stem cells. **a** Schematic for isolation and analysis of primary tumor cells (green), dormant residual tumor cells (red), recurrent tumor cells (blue), and stromal cells from recurrent tumors (yellow). **b** Dot plot showing first (PC1) and second (PC2) principal components from analysis of gene expressed in sorted primary tumor cells, dormant residual tumor cells, and recurrent tumor cells from *HER2/neu-Prim1* (left) and *Wnt1-Prim1* (right) models. **c**, **e**, **g** Bar graphs showing mean expression level and standard error of the mean (SEM) in primary tumor cells (green), dormant residual tumor cells (red), recurrent tumor cells (blue), and stromal cells from recurrent tumors (yellow), for genes associated with candidate dormancy regulatory genes (**c**), or epithelial and mesenchymal phenotypes (**e**, **g**) for HER2/neu (**e**) or Wnt1 (**g**) models. **d** Forest plot showing hazard ratio of recurrence-free survival (RFS) for patients with high dormancy signature scores across seventeen human primary breast cancer data sets. **f**, **h** Dot plot showing rank-ordered log2 fold-change of gene expression in residual tumor cells vs. all other cells for *HER2/neu* (**f**) and *Wnt1* (**h**) models. Genes from expression signatures for normal mammary stem cells (left) or neoplastic mammary stem cells (right), which also met criteria for differential expression in their respective datasets (|fold-change| > 1.5, FDR < 0.1), are indicated in red. Genes from these signatures that did not meet criteria for differential expression are not listed. *p value residual tumor cells vs. primary tumor cells < 0.05, **p value residual tumor cells vs. recurrent tumor cells < 0.05

To assess the molecular phenotype of residual tumor cells and identify candidate mechanisms potentiating cellular dormancy in the context of oncogenic pathway inhibition, we performed gene expression profiling on purified dormant residual tumor cells. H2B-eGFP-labeled *HER2/neu-Prim1* or *Wnt1-Prim1* tumor cells were used to generate orthotopic primary tumors, 28-day MRLs, and recurrent tumors. GFP+ tumor cells were isolated from enzymatically digested tumors and MRLs using fluorescence-activated cell sorting and subjected to expression profiling, along with GFP-negative stromal cells isolated from recurrent tumors in the HER2/neu model (Fig. 4a).

Principal component analysis revealed that residual tumor cells exhibit a gene expression pattern that is unique compared to either primary or recurrent tumor cells (Fig. 4b). To identify functionally related groups of genes, DAVID gene ontology analysis was performed on genes differentially expressed in residual tumor cells compared to both primary and recurrent tumor cells from the same model.

Genes downregulated in residual tumor cells were dramatically enriched for cell cycle-related and ribosomal gene sets in both the *HER2/neu* and *Wnt1* models (Additional file 4: Table S1). Consistent with our observation that residual tumor cells are quiescent, transcripts for cyclins and cyclin-dependent kinases were downregulated as much as 90% in residual tumor cells (Additional file 5: Fig. S4a, b). Unsupervised hierarchical clustering of gene expression levels for ribosomal proteins revealed broad downregulation in residual tumor cells compared to primary or recurrent tumor cells (Additional file 5: Fig. S4c). As ribosomal biogenesis can be regulated by mTOR activity [68], we quantified mTOR pathway activity using gene expression-based computational methods [51]. This revealed significant downregulation of mTOR pathway activity in both *HER2/neu* and *Wnt1* residual tumor cells compared to their corresponding primary or recurrent tumor cells (Additional file 5: Fig. S4d). Conversely, consistent with the abundant desmoplastic stroma identified within MRLs (Additional file 1: Fig. S1), differentially upregulated transcripts in residual tumor cells were enriched for secretory and ECM gene sets, including genes such as *fibronectin* (Additional file 6: Table S2).

Previous analyses of head and neck squamous cell carcinoma (HNSCC) cell lines that proliferate in vitro, but do not proliferate upon seeding metastatic sites in vivo, have suggested downregulation of uPAR, or upregulation of TGFβ-II/TGFβR-III signaling, as a cause of a low ERK:p38 signaling ratio, which in turn upregulates the transcriptional repressor DEC2 and downregulates the transcriptional activator FOXM1 [69, 70]. Consistent with these findings in mouse models for dormancy induced by microenvironmental cues, we found that Dec2 exhibited the greatest upregulation among all transcription factors, and that FoxM1 was significantly downregulated, in residual tumor cells in both the *HER2/neu* and *Wnt1* models (Fig. 4c). Furthermore, we found that dormant residual tumor cells in vivo exhibit increased expression of *Tgfbr3* (TGFβR-III), as well as its ligand *Tgfb2* (TGFβ-II), and markedly decreased expression of *Plaur* (uPar) compared to both primary and recurrent tumor cells (Fig. 4c).

Additionally, we found that *Thsb1* (Thrombospondin-1) expression in residual tumor cells in both the *HER2/neu* and *Wnt1* models was elevated ~ 3-fold compared to primary or recurrent tumor cells (*p* values < 0.01; Fig. 4c). Thsb1 has been implicated in promoting dormancy in isolated breast cancer cells that do not form metastases upon seeding metastatic sites in xenograft models [71]. Together, these findings reveal that dormant residual tumor cells surviving oncogenic pathway inhibition exhibit molecular features similar to those observed in models of isolated DTCs in which dormancy is induced by microenvironmental cues.

In aggregate, expression profiling experiments confirm that residual tumor cells surviving oncogene inhibition are dormant and reveal that these cells exist in a state that is unique compared to primary or recurrent tumor cells, and that is characterized by decreased proliferation, ribosomal biogenesis, and mTOR activity, as well as increased expression of ECM proteins. These findings further suggest that molecular mechanisms regulating dormancy induced by targeted blockade of an oncogenic pathway may share similarities with those regulating dormancy induced by microenvironmental cues, and that these mechanisms may be conserved in dormant tumor cells derived from tumors driven by distinct oncogenic pathways, as well as different cancer types.

### A mouse signature for dormant residual tumor cells predicts recurrence-free survival in breast cancer patients

To evaluate the clinical relevance of these mouse models of dormant MRD, we reasoned that elevated expression of genes associated with dormant residual disease in mice might be associated with prolonged latency periods prior to recurrence (i.e., increased recurrence-free survival) in breast cancer patients. To test this hypothesis, we generated a dormancy signature from genes that were differentially up- or downregulated in dormant mouse residual tumor cells compared to primary and recurrent tumor cells, in both the *HER2/neu* and *Wnt1* tumor models.

Meta-analysis of human recurrence-free survival (RFS) data representing ~ 4400 breast cancer patients revealed that patients whose tumors displayed higher levels of this dormancy signature exhibited markedly decreased rates of tumor recurrence (HR = 0.49, *p* = 4.8E-35) (Fig. 4d). This mouse dormancy signature retained its prognostic value after adjusting for proliferation in a multivariate model, indicating that its association with RFS was not simply due to proliferation-associated genes (data not shown).

The observation that a gene expression signature specific for dormant residual tumor cells in mice is robustly associated with an increased latency to recurrence in breast cancer patients further supports the clinical relevance of these mouse models for dormant MRD. This finding is particularly notable given that most human tumors in these data sets are ER+ and recurred at distant (i.e., metastatic) sites, whereas the mouse models from which this dormancy signature was derived are ER-negative and recurred locally.

### Dormant residual *HER2/neu*, but not *Wnt1*, tumor cells exhibit a mesenchymal phenotype

While residual disease in breast cancer patients has been reported to be enriched for a mesenchymal phenotype following neoadjuvant chemotherapy [72], whether residual tumor cells are enriched for a mesenchymal phenotype following targeted inhibition of oncogenic pathways is unclear. Analysis of gene expression in *HER2/neu* tumor cells revealed that residual tumor cells exhibit increased expression of transcripts associated with a mesenchymal phenotype and decreased expression of transcripts associated with an epithelial phenotype, compared to primary tumor cells (Fig. 4e).

In contrast, residual tumor cells in the *Wnt1* model did not exhibit increased expression of mesenchymal markers and instead displayed increased expression of the basal epithelial marker *Krt14* (CK14) along with luminal epithelial markers *Epcam* and *Cdh1* (Fig. 4g). Consistent with their expression in both myoepithelial and mesenchymal cells, expression of *Pdgrfb* and *Acta2* were increased in residual tumor cells in both the *HER2/neu* and *Wnt1* models (Fig. 4e, g). Together, these data suggest that dormant residual disease in the *HER2/neu* model may be enriched for tumor cells that have undergone epithelial-to-mesenchymal transition (EMT), whereas dormant residual disease in the *Wnt1* model may be enriched for tumor cells with a basal epithelial phenotype.

To confirm these findings, we performed IF for luminal (CK8) and basal (CK14) epithelial proteins on H2B-eGFP-labeled primary tumors and MRLs. As anticipated, primary tumor cells in both the *HER2/neu* and *Wnt1* models expressed CK8, and a subset of Wnt1 tumor cells expressed CK14 (Additional file 7: Fig. S5a, b). In contrast, residual *HER2/neu* tumor cells did not express CK8, whereas residual *Wnt1* tumor cells continued to express CK8 and CK14 (Additional file 7: Fig. S5a, b). IF staining using a pan-cytokeratin antibody, as well as seven additional epithelial proteins including CK5, CK18, CK19, EpCAM, E-Cadherin, P-Cadherin, and p63, confirmed that residual *HER2/neu* tumor cells generally lacked expression of epithelial markers, suggesting that they had undergone an EMT (Additional file 8: Fig. S6).

Flow cytometry for epithelial (CD24) and mesenchymal (PDGFR-β or CD49e) proteins confirmed the epithelial nature of orthotopic primary H2B-eGFP-labeled *HER2/neu* tumors, and further revealed rare populations of mesenchymal CD24–PDGRF-(beta)+ and CD24–CD49e+ primary tumor cells that were dramatically enriched in MRLs following HER2/neu downregulation (Additional file 7: Fig. S5d-g).

Together, these findings indicate that HER2/neu-induced primary tumor cells are predominantly epithelial, but contain a small population of mesenchymal cells that is dramatically enriched among cells that survive HER2/neu downregulation and that persist in a dormant state within MRLs. In contrast, both primary and residual tumor cells from Wnt1-induced tumors express epithelial markers characteristic of luminal and myoepithelial cells.

### Residual micrometastatic tumor cells recapitulate features of local residual disease

As distant recurrence is the principal cause of mortality from breast cancer, we examined micrometastatic disease in the lung following oncogenic pathway inhibition in *MTB;TetO-HER2/neu*;*TetO-TurboCre;Rosa26-lox-stop-lox-YFP* (*MTB;TetO-HER2/neu;TTC;rYFP*) mice. Doxycycline-treated mice of this genotype induce both HER2/neu and Cre and develop lineage-marked mammary adenocarcinomas in which all tumor cells express YFP. Fluorescently marked primary tumors in mice were then enzymatically digested to a single-cell suspension and used to generate orthotopic primary tumors in *nu/nu* recipient mice that subsequently developed YFP+ lung metastases. Lung metastases in recipient mice on doxycycline were compared to residual metastatic foci in mice from which doxycycline had been withdrawn for 28 days.

**Fig. 5:**
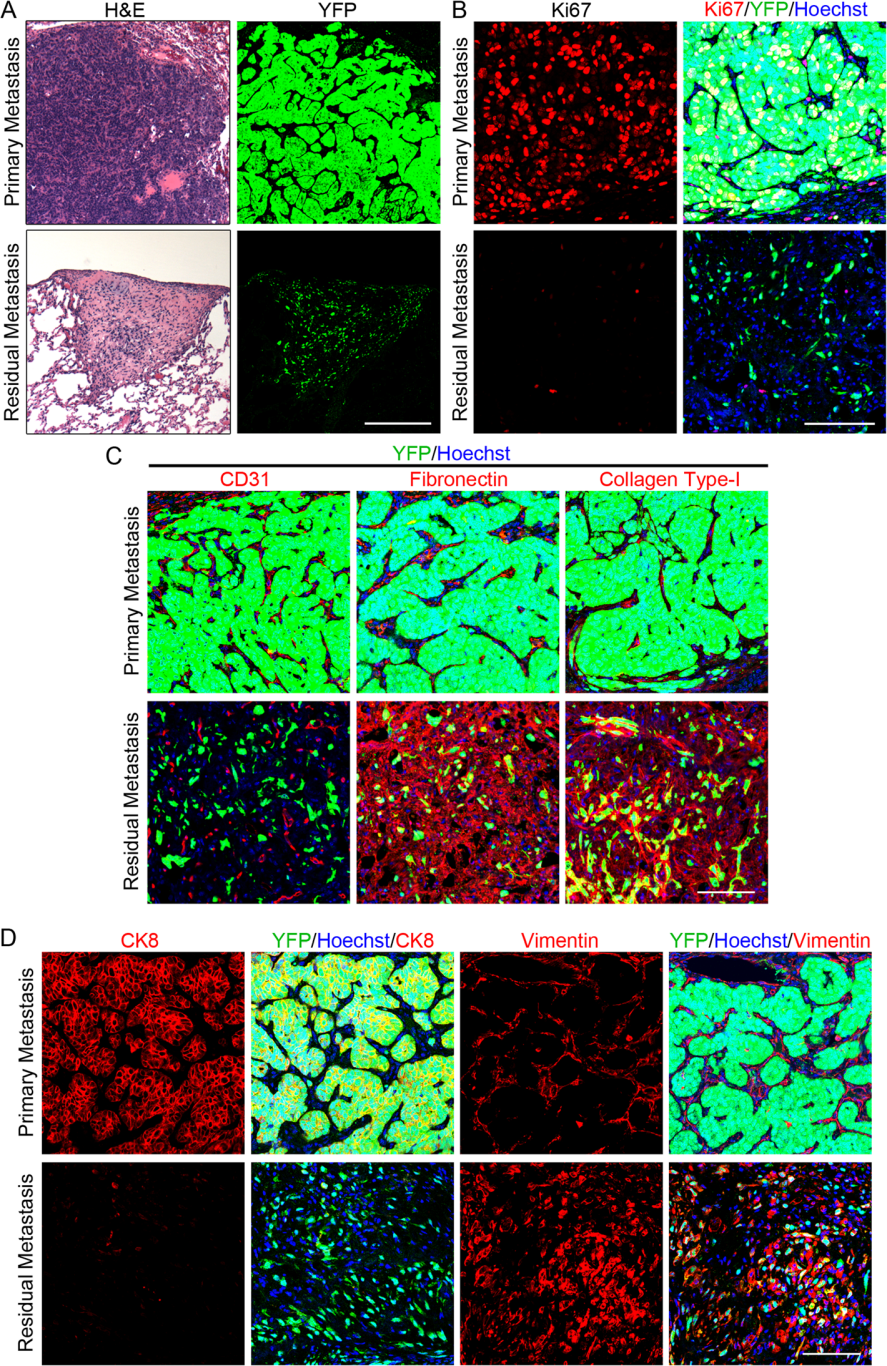
Residual metastases exhibit cellular dormancy. **a–d** Lung metastasis in *MTB;TetO-HER2/neu;TTC;rYFP* mouse on doxycycline (top) or residual lung metastasis following doxycycline withdrawal (bottom). **a** H&E-stained sections (left) or YFP fluorescence microscopy (right). **b** IF for Ki67 (left) or Ki67, YFP and Hoechst 33258 (right). **c** IF for YFP, Hoechst 33258, and CD31 (left), fibronectin (center) or collagen type-I (right). **d** IF for CK8 (left), CK8, YFP, and Hoechst 33258 (center-left), vimentin (center-right), or vimentin, YFP, and Hoechst 33258 (right). Scale bars (**a**) 250 μm and (**b–d**) 100 μm

This analysis revealed that residual metastatic foci strongly resembled local residual disease. First, while lung metastases in mice on doxycycline displayed well-delineated epithelial and stromal compartments, tumor cells in residual metastatic foci were scattered throughout an eosinophilic stroma (Fig. 5a). Second, the majority of metastatic tumor cells in mice on doxycycline were Ki67+, whereas metastatic tumor cells in residual foci were uniformly Ki67-negative (Fig. 5b). Third, residual metastatic foci were well-vascularized and highly desmoplastic, in contrast to lung metastases in mice maintained on doxycycline (Fig. 5c). Finally, tumor cells within residual metastatic foci in the lung did not express the epithelial marker CK8 and acquired expression of the mesenchymal marker vimentin, consistent with an EMT (Fig. 5d). Together, these data reveal that residual metastatic tumor cells surviving oncogenic pathway inhibition display cellular dormancy, EMT, and a desmoplastic, vascularized stroma similar to that observed in dormant local residual disease.

### Human breast cancer cells exhibit cellular dormancy following targeted therapy

**Fig. 6:**
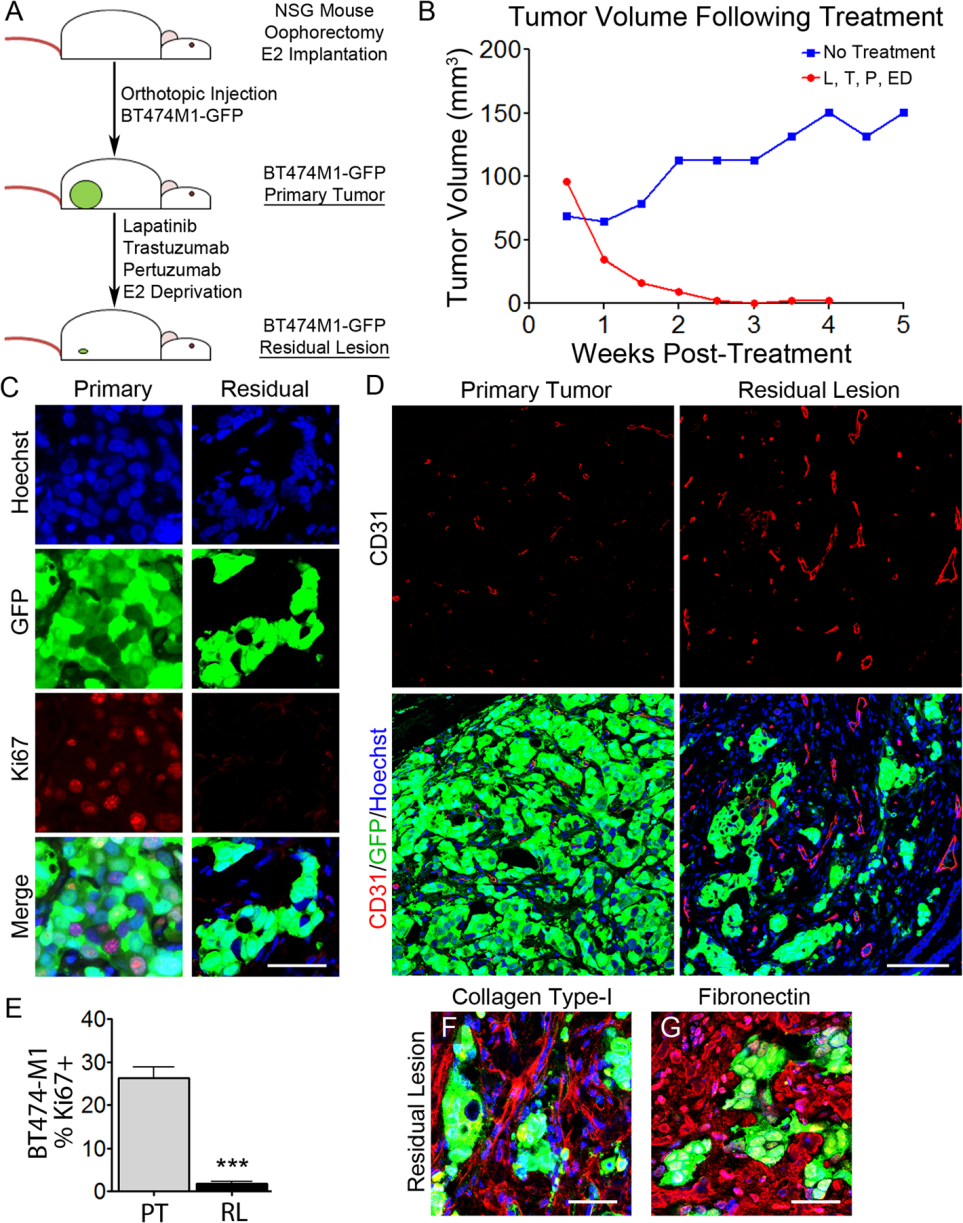
Human breast cancer xenografts exhibit quiescence following targeted therapy. **a** Schematic of generation of xenograft primary tumors and MRLs following targeted therapy. **b** Tumor volumes in mice treated with vehicle control or the combination of lapatinib (L), trastuzumab (T), pertuzumab (P), and estrogen deprivation (ED). **c** IF staining for Ki67, GFP, and Hoechst 33258 on sections of BT474M1-GFP primary tumor (left), or residual disease (right). **d** IF staining for CD31, GFP, and Hoechst 33258, on sections of BT474M1-GFP primary tumor (left) or residual lesion (right), showing CD31 alone (top), or CD31 in combination with GFP and Hoechst 33258 (bottom). **e** Quantification of GFP-labeled BT474M1 tumor cells staining positive for Ki67 from **c**, on sections from primary tumors (PT) or residual lesions (RL). **f**, **g** IF staining for collagen type-I (**f**) and fibronectin (**g**) on sections of orthotopic BT474M1-GFP residual lesions. ***p value vs. primary tumor cells < 0.001. Scale bars (**c**, **f**, **g**) 50 μm and (**d**) 100 μm

To address the possibility that residual human breast cancer cells surviving oncogenic pathway inhibition might also be dormant, we generated orthotopic xenografts from GFP-labeled BT474-M1 breast cancer cells. Following the development of 1 cm primary tumors, a cohort of mice was treated with triplet anti-HER2 therapy coupled with anti-estrogen receptor (ER) therapy (Fig. 6a). Analogous to tumors in *MTB;TetO-HER2/neu* mice following oncogene downregulation, treatment of orthotopic BT474-M1 tumors with targeted agents resulted in their rapid regression to a non-palpable state, whereas untreated tumors continued to grow (Fig. 6b).

Consistent with our observations in *HER2/neu* and *Wnt1* transgenic mouse models, residual BT474-M1 lesions contained scattered GFP+ tumor cells that were quiescent, with only ~ 2% expressing Ki67 (Fig. 6c, e). In contrast, greater than 25% of untreated primary tumor cells were Ki67+. Furthermore, IF for CD31 revealed a rich vasculature within BT474-M1 residual lesions (Fig. 6d) along with dense deposition of fibronectin and type I collagen surrounding residual tumor cells, indicative of a desmoplastic ECM (Fig. 6f, g). Together, these findings suggest that quiescent residual tumor cells residing in a well-vascularized, desmoplastic microenvironment may be a general feature of residual disease following oncogenic pathway inhibition. Further, they provide a conceptual link between murine residual disease following oncogene downregulation and residual human breast cancer cells following targeted therapy.

### Dormant residual tumor cells express a mammary stem cell signature

As tumor cells that undergo EMT have been suggested to acquire properties of normal mammary stem cells [73], and since residual tumor cells surviving HER2/neu (but not Wnt1) downregulation exhibited features of EMT, we wished to evaluate whether *HER2/neu* or *Wnt1* residual tumor cells exhibit gene expression profiles similar to those of mammary stem cells.

Enrichment analysis was performed by comparing genes reported to be upregulated in normal mammary stem cells [74] with genes differentially expressed in *HER2/neu* or *Wnt1* residual tumor cells compared to primary and recurrent tumors cells. This analysis revealed that genes associated with human mammary stem cells were expressed at significantly higher levels in dormant residual tumor cells compared to all other tumor cell types in both the *HER2/neu* and *Wnt1* models (Fig. 4f, h; *p* value *HER2/neu* = 2.34E-27; *p* value *Wnt1* = 1.72E−14).

Tumor-initiating cells (TICs) identified in some model systems have been suggested to exhibit features of mammary stem cells [75]. Enrichment analysis performed for genes associated with breast cancer stem-like cells [76] revealed that residual tumor cells in both the *HER2/neu* and *Wnt1* models upregulated genes associated with human breast cancer TICs (Fig. 4f, h; *p* value *HER2/neu* = 0.001; *p* value *Wnt1* = 2.23E−12).

Collectively, these findings suggest that dormant tumor cells that survive HER2/neu or Wnt1 downregulation exhibit gene expression profiles resembling those of normal mammary stem cells and tumorigenic breast cancer cells.

### Residual disease and enrichment for tumor-initiating cells

**Fig. 7:**
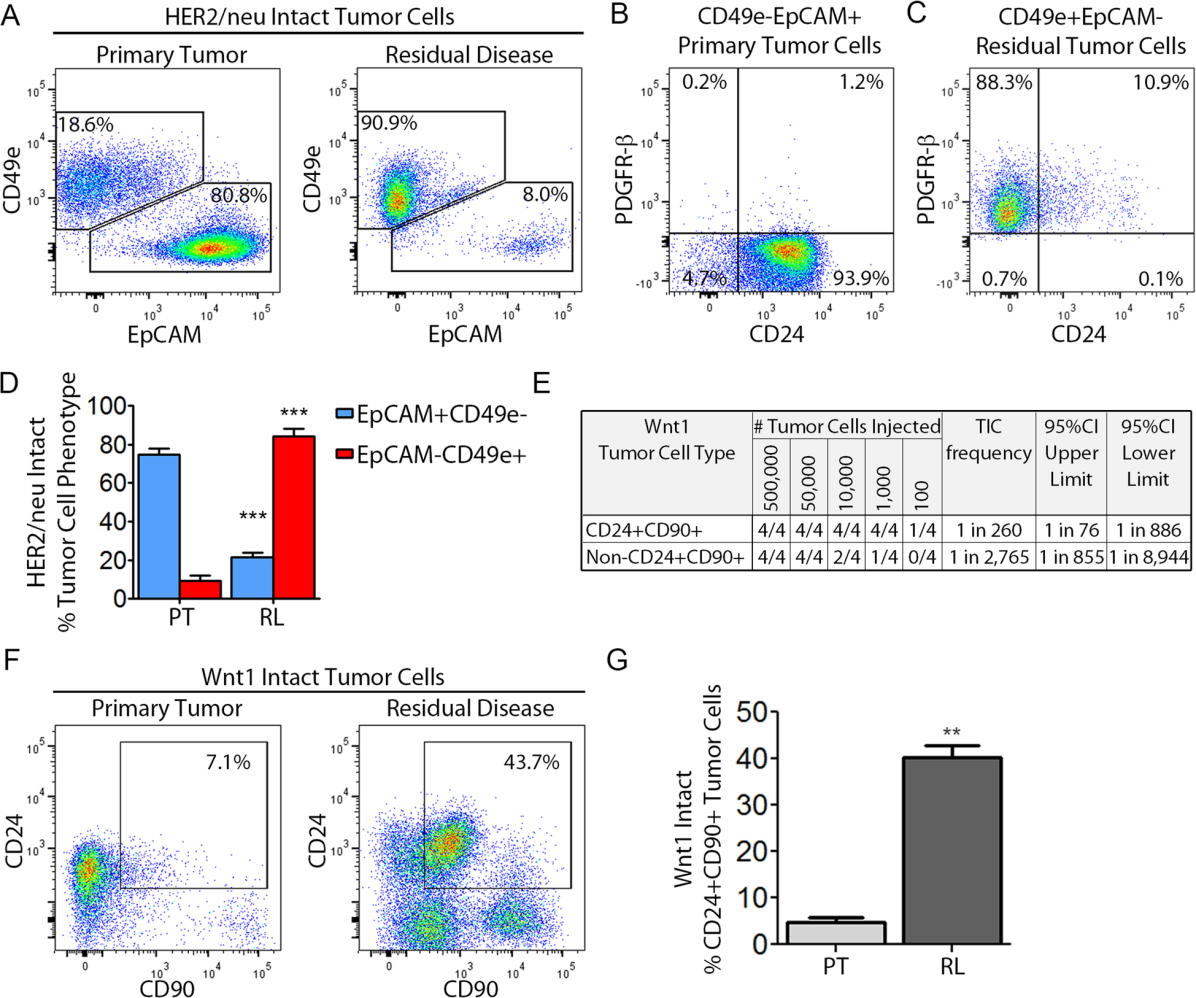
Residual disease is enriched for rare tumor cell subpopulations present in primary tumors. **a** Flow cytometry for EpCAM and CD49e on YFP+CD45−DAPI− singlet tumor cells in *MTB;TetO-HER2/neu;TTC;rYFP* primary tumor cells (left) or dormant residual tumor cells (right). **b**, **c** Flow cytometry on populations in (**a**) for CD24 and PDGFR-β expression on CD49e−EpCAM+ primary tumor cells (**b**) or CD49e+EpCAM− residual tumor cells (**c**). **d** Quantification of percent CD49e−EpCAM+ or CD49e+EpCAM− tumor cells in primary tumors or residual lesions, using gates from (**a**), in *MTB;TetO-HER2/neu;TTC;rYFP* mice. **e** Limiting dilution assay of CD24+Thy1+ tumor cells, or non-CD24+Thy1+ tumor cells, from *MTB;TetO-Wnt1;TTC;rYFP* primary tumors. **f** Flow cytometry for CD24 and Thy1 expression on YFP+CD45−DAPI− singlet tumor cells in *MTB;TetO-Wnt1;TTC;rYFP* primary tumor cells (left) or residual tumor cells (right). **g** Quantification of percent CD24+Thy1+ or non-CD24+Thy1+ tumor cells in primary tumors or residual lesions, using gates from **f**, in *MTB;TetO-Wnt1;TTC;rYFP* mice. **p value vs. primary tumor (PT) < 0.01, ***p value vs. PT < 0.001

Our observations that residual tumor cells are capable of giving rise to recurrent tumors, possess mesenchymal features, and are enriched for transcripts associated with normal mammary stem cells and tumorigenic breast cancer cells, suggested that residual tumor cells might be functionally enriched for TICs. To test this hypothesis in a model with minimal ex vivo manipulation, we used *MTB;TetO-HER2/neu;TTC;rYFP* mice to isolate fluorescently labeled tumor cells from autochthonous tumors that had not been cultured in vitro. To control for genetic heterogeneity between different donor tumors, we used primary tumors from *MTB;TetO-HER2/neu;TTC;rYFP* mice to generate matched pairs of syngeneic orthotopic primary tumors and residual lesions. Consistent with our findings in the *HER2/neu-Prim1* orthotopic model, uncultured orthotopic primary tumors derived from *MTB;TetO-HER2/neu;TTC;rYFP* mice were predominantly epithelial (CD24+EpCAM+CD49e−PDGFR-β−), whereas residual lesions were enriched for tumor cells with mesenchymal-like properties (CD24−EpCAM−CD49e+PDGFR−β+) (Fig. 7a–d).

YFP+CD45−DAPI− tumor cells from these syngeneic pairs of primary tumors and residual lesions were used to perform limiting dilution experiments in *nu/nu* mice maintained on doxycycline to quantify TIC frequency in primary tumors and residual lesions generated from the same intact donor tumor. This revealed that TICs were not enriched in residual tumor cells compared to primary tumor cells generated from the same donor tumor (Additional file 9: Table S3).

We next asked whether the lack of enrichment for TICs in *HER2/neu* residual tumor cells was also true for Wnt1 residual tumor cells. As CD24+Thy1+ mammary tumor cells from *MMTV-Wnt1* mice have been reported to possess TIC properties [77], we first performed limiting dilution experiments to ask whether CD24+Thy1+ primary tumor cells from *MTB;TetO-Wnt1;TTC;rYFP* mice were enriched for TICs. Consistent with prior reports, CD24+Thy1+ tumor cells were enriched ~ 10-fold for TICs compared to non-CD24+Thy1+ tumor cell subsets (Fig. 7e). Furthermore, we found that residual tumor cells in *MTB;TetO-Wnt1;TTC;rYFP* mice were enriched ~ 8-fold for CD24+Thy1+ tumor cells compared to primary tumors (Fig. 7f, g).

Despite this, limiting dilution studies in *nu/nu* mice on doxycycline using YFP+CD45−DAPI− tumor cells isolated from syngeneic pairs of orthotopic primary tumors and residual lesions derived from individual donor tumors arising in *MTB;TetO-Wnt1;TTC;rYFP* mice revealed that residual *Wnt1* tumor cells exhibited lower TIC frequencies than primary tumor cells (Additional file 10: Table S4). Together, the above findings suggest that residual tumor cells are not enriched for TICs compared to primary tumor cells in either the *HER2/neu* or *Wnt1* mammary tumor models when assayed in *nu/nu* mice maintained on doxycycline.

In light of our observation that mice bearing fully regressed *HER2/neu* or *Wnt1* mammary tumors eventually develop spontaneous tumor recurrences when maintained off doxycycline [17, 18], we considered the possibility that residual tumor cells might be enriched for TICs capable of giving rise to recurrent, rather than primary, tumors. To address this possibility, we generated orthotopic primary tumors and residual lesions from H2B-eGFP *HER2/neu-Prim1* tumor cells. TIC frequencies were assessed in primary and residual tumor cells by limiting dilution following injection into cohorts of *nu/nu* mice maintained either on or off doxycycline.

Consistent with our observations in intact tumor-bearing *MTB;TetO-HER2/neu;TTC; rYFP* mice and in mice bearing orthotopic *HER2/neu-Prim1* primary tumors, we did not observe enrichment for TICs among residual tumor cells when injected into mice on doxycycline. In contrast, when injected into *nu/nu* mice not on doxycycline, we observed a ~ 7-fold increase in TIC frequency among residual tumor cells compared to primary tumor cells (Additional file 11: Table S5). These findings suggest that HER2/neu residual disease may be enriched for cells capable of giving rise to recurrent, but not primary, tumors.

## Discussion

Defining the biology of minimal residual disease is an essential goal with important clinical implications for the prevention of recurrent cancers and improving cancer outcomes. Using genetically engineered mouse models for human breast cancer driven by two different oncogenic pathways, we report in vivo evidence that tumor cells surviving targeted therapy exist in a state of cellular dormancy and do so despite the presence of a robust functional vasculature and irrespective of the presence or absence of adaptive immunity. In an analogous manner, we also found evidence for cellular dormancy in mice bearing micrometastatic disease in the lungs following oncogenic pathway inhibition and in human breast cancer xenografts treated with targeted therapies. Together, our findings indicate that residual tumors cells surviving targeted inhibition of a dominant oncogenic pathway exist in a state of cellular dormancy at both local and distant sites, and that dormancy in this context is not a consequence of angiogenic insufficiency nor adaptive immunity. Rather, our observations suggest that dormancy may be a conserved response to targeted therapy independent of cell type of origin or oncogenic pathway inhibited, and that the mechanisms underlying dormancy at local and distant sites may be related.

Our findings that a tumor dormancy signature derived from mouse residual tumor cells is strongly associated with risk of both early and late recurrence in a meta-analysis of RFS in ~ 4400 breast cancer patients, and retains its prognostic value after adjusting for proliferation in a multivariate model, suggests that features of dormancy observed in mouse models are recapitulated in patients and supports the clinical relevance of these GEM models for tumor dormancy. This finding is particularly striking given that the mouse models from which this signature was derived are ER-negative and model local recurrence, whereas the patient data sets queried principally reflect ER+ breast cancers that recurred at distant (i.e., metastatic) sites. These observations suggest that the biology of these GEM models is neither specific for, nor restricted to, a particular breast cancer subtype, nor for local as opposed to distant recurrence.

Additional evidence supports the relevance of these mouse models for understanding dormancy and recurrence in patients. For example, the above mouse dormancy signature shows similarities with signatures derived from bone marrow DTCs in prostate cancer patients as well as human xenograft models for head and neck, prostate, and breast cancer dormancy induced by the microenvironment [57, 70, 78–80], suggesting that mouse residual tumor cells surviving targeted therapy may be biologically similar to DTCs in patients. Furthermore, the survival of residual tumor cells in the mouse mammary gland following oncogene downregulation parallels patients who receive neoadjuvant chemotherapy, but do not achieve a pathologic complete response; in both mice and humans, survival of residual tumor cells in the mammary gland is associated with an increased risk of distant recurrence.

Beyond the findings presented here, prior functional interrogation of these GEM models has identified multiple pathways that contribute to tumor recurrence in mice, each of which is associated with risk of distant relapse in patients, in the direction predicted by studies in mice, and in a manner that is not restricted to ER+ or ER-negative breast cancers [18, 23–26]. It is also interesting to note that recurrent tumors in *MTB;TetO-HER2/neu* mice typically lack HER2 overexpression, such that recurrence is driven by the activation of alternate pathways [18, 23–25, 81]. This is paralleled by clinical observations that HER2+ primary breast cancers in patients frequently give rise to HER2-negative residual disease [82–84] and recurrent tumors [84]. In addition, local recurrence in patients is strongly associated with an increased risk of distant relapse and mortality [27–30], and the timing of local and distant relapse following surgery are similar [31], suggesting that the mechanisms by which tumor cells survive and recur—whether local or distant—are related. Finally, expression profiles of residual tumor cells from *HER2/neu* and *Wnt1* GEM models are strikingly similar to each other, suggesting that residual tumor cell biology reflects conserved properties and is not specific to the specific oncogenic pathway that induced the tumor. In aggregate, these findings support the broad clinical relevance of GEM models for tumor dormancy and recurrence, and suggest that they are likely informative for the biology of residual tumor cells that survive selective pressures imposed by either targeted therapy or the microenvironment, and in a manner that is not restricted to ER+ versus ER-negative human breast cancers or to local versus distant sites of recurrence.

Through expression profiling of in vivo-purified residual tumor cells along with genetically matched primary and recurrent tumor cells, we determined that dormant residual tumor cells that survive targeted therapy in mice share expression features with residual tumors cells from human xenograft models for head and neck cancers, as well as breast cancers, in which dormancy occurs in response to microenvironmental cues. This included decreased expression of *uPAR* as well as increased expression of *TGFβ-II/TGFβR-III*, each of which has been implicated in downregulating the ERK:p38 signaling ratio [69, 70], downregulating *FOXM1*, and upregulating *DEC2/BHLHe41*—each of which we also observed in both the *HER2/neu* and *Wnt1* models, as well as increased expression of Thrombospondin-1 [71]. In aggregate, these findings suggest that tumor cells surviving targeted therapies exist in a state of cellular dormancy resembling that induced by microenvironmental cues.

Notably, expression profiling of in vivo-purified residual tumor cells also revealed that residual tumor cells exist in a unique state that bears little resemblance to actively growing primary or recurrent tumor cells. This has important implications for both the detection and treatment of residual disease. Given the unique phenotype of residual tumor cells, it seems likely that detection approaches based on biomarkers expressed on primary or recurrent tumor cells may fail to detect some—or possibly many—residual tumor cells. In an analogous manner, therapeutic approaches predicated on increased proliferative or metabolic activity commonly associated with actively growing tumor cells may fail to impact the long-term survival or regrowth of dormant residual tumor cells.

Although recurrent tumors must logically arise from the reservoir of DTCs that survive therapy, and while the presence of DTCs in bone marrow following treatment is an independent prognostic factor for recurrence-free survival in multiple cancer types, little preclinical or clinical evidence exists demonstrating a precursor-product relationship between the two. As such, it is possible that dormant MRD in bone marrow is correlated with systemic disease burden, but that these cells do not themselves give rise to recurrent tumors. In light of this uncertainty, our observation that residual tumor cells surviving targeted therapy reside in a dormant state, yet remain capable of giving rise to recurrent tumors, suggests that dormancy may indeed represent a therapeutic target for preventing tumor recurrence following targeted therapy. Beyond the stochastic kinetics of tumor recurrence, which implies the existence of a dormant phase, we also failed to observe clusters of BrdU-positive tumor cells even after 28 days of BrdU labeling, as might have been expected if the clonal expansion of rare cells that failed to enter a dormant state following oncogene downregulation was responsible for giving rise to recurrent tumors. In this regard, it is notable that gene expression profiles for residual disease in the *HER2/neu* and *Wnt1* models were remarkably similar despite dramatic phenotypic differences between primary tumors induced by these two pathways. This suggests that therapeutic strategies targeting properties unique to dormant residual disease may be tractable, particularly for eradicating tumor cell populations that survive treatment with targeted therapies.

Interestingly, dormant residual tumor cells in both the *Wnt1* and *HER2/neu* mouse models were enriched for expression signatures associated with normal, as well as neoplastic, human mammary stem-like cells. Residual tumor cells in each of these models were also enriched for phenotypes reported to be associated with TICs—EMT, in the case of *HER2/neu*, and a CD24+Thy1+ cell surface phenotype, in the case of *Wnt1*. Despite these suggestive phenotypic traits, functional studies revealed that residual tumor cells were not enriched for primary tumor TICs. These findings suggest that phenotypes of TICs may differ between primary tumors and MRD and imply that caution should be exercised in attempting to infer functional properties of residual tumor cells based upon cell surface phenotypes defined in TICs in primary tumors.

Although residual tumor cells failed to show enrichment for TICs compared to primary tumor cells in the setting of oncogene activation, tumor cells isolated from residual lesions were enriched for TICs capable of giving rise to recurrent tumors in the setting of oncogene inhibition. This finding suggests the possibility that tumor evolution, from primary to recurrent, may be driven by the emergence and selection of distinct tumorigenic cell populations. This possibility is supported by our prior finding that recurrent tumors in *MTB;TetO-HER2/neu* mice exhibit a phenotype distinct from primary tumors [18] and by subsequent reports in patients indicating that recurrent tumors may exhibit a phenotype distinct from primary tumors [72, 82, 85].

Overall, despite the association between EMT, stem-like cells, and resistance to therapy reported in some experimental contexts, our findings that *HER2/neu* residual disease is enriched for cells with an EMT-like phenotype whereas *Wnt1* residual disease is not, and that neither *HER2/neu* nor *Wnt1* residual disease is enriched for primary tumor TICs, suggest that the properties of TICs, mammary stem cells, and EMT are separable, at least in some contexts. Consistent with this, recent observations in circulating tumor cells suggest that residual tumor cells with an epithelial, rather than mesenchymal, phenotype may be enriched in certain therapeutic settings [86].

## Conclusions

In summary, our observations reveal that residual cancer cells surviving targeted therapy exist in a state of cellular dormancy at both local and distant sites that is not due to angiogenic insufficiency or adaptive immunity. Residual tumor cells exhibit a unique gene expression profile compared to primary or recurrent tumor cells that is conserved across mouse models for human breast cancer driven by different oncogenes, and with tumor cells in which dormancy has been induced by microenvironmental cues. Dormant residual tumor cells retain the ability to re-enter the cell cycle and give rise to recurrent tumors after extended latency periods and possess a gene expression signature that overlaps with those of normal and neoplastic mammary stem cells, and is strongly associated with recurrence-free survival in breast cancer patients. Further, TIC populations that underlie primary tumorigenesis may be distinct from those which give rise to recurrence following therapy, further highlighting the importance of elucidating the unique biology of recurrent cancer. In aggregate, our findings suggest that cellular dormancy following targeted therapy may contribute to cancer recurrence, and that therapeutic strategies aimed at selectively targeting this state may prevent tumor relapse.

## Supplementary Information

**Additional file 1: **Figure S1. Kinetics of tumor recurrence suggest a latent phase. (a-d) Kaplan-Meier curves showing recurrence-free survival (RFS) for (a) *MTB;TetO-HER2/neu* intact, (b) *HER2/neu-Prim1* orthotopic, (c) *MTB;TetO-Wnt1* intact, and (d) *Wnt1-Prim1* orthotopic models. (e) Recurrent tumor growth curves from *MTB;TetO-HER2/neu* orthotopic tumors with different recurrence latencies. (f) Tumor growth curves following doxycycline re-administration to intact *MTB;TetO-HER2/neu* mice harboring regressed primary tumors that had not spontaneously recurred.

**Additional file 2: **Figure S2. ECM protein expression in minimal residual lesions. (a-d) IF staining showing Hoechst 33258 (blue), H2B-eGFP (green) and either collagen type-I (red, a, c) or fibronectin (red, b, d) on H2B-eGFP-labeled orthotopic *HER2/neu-Prim1* MRLs (a, b) or normal mammary ducts (c, d), labeled to show duct epithelial cells (D), or lumen (Lu). Scale bar 50 μm for all images.

**Additional file 3 **CD31 co-localizes with intravenously injected lectin-AF647. (a-f) Fluorescence microscopy for Hoechst 33258 (blue, a), IF for CD31 (red, b), H2B-eGFP (Green, d), lectin-AF647 (Yellow, eE), merge of CD31 and Lectin-AF647 (c), or merge of all channels (f) on H2B-eGFP-labeled orthotopic *HER2/neu-Prim1* MRL. Scale bar 100 μm for all images.

**Additional file 4:.** Table S1. Gene sets down-regulated in dormant residual tumor cells. Clusters of SP-PIR Keywords identified by DAVID functional ontology analysis of genes down-regulated in dormant residual tumor cells compared to all other cell types (false discovery rate (FDR) < 0.1, fold-change (FC) > 1.5-fold).

**Additional file 5: **Figure S4. Analysis of gene expression data for mitosis-related genes and mTOR pathway activity. (a, b) Expression of all differentially expressed cyclins and cyclin-dependent kinases in HER2/neu (a) and Wnt1 (b) models. (c) Relative expression of all ribosomal proteins, and unsupervised hierarchical clustering for HER2/neu (left) and Wnt1 (right) tumors. (d) Expression of mTOR signature in primary tumor (PT), residual lesion (RL), recurrent tumor (RT) and recurrent tumor stromal cells (RS), for individual (circles) and mean (black line) of pathway activity for HER2/neu (left) and Wnt1 (right) tumors. *p*-value vs. RL for mTOR signature of HER2/neu: PT < 1.0E-6, RT = 2.1E-3, RS = 4.6E-3; Wnt1: PT < 1.0E-6, RT < 1.0E-4.

**Additional file 6:.** Table S2. Gene sets up-regulated in dormant residual tumor cells. Clusters of SP-PIR Keywords identified by DAVID functional ontology analysis of genes up-regulated in dormant residual tumor cells compared to all other cell types (FDR < 0.1, FC > 1.5-fold).

**Additional file 7: **Figure S5. HER2/neu-Prim1 residual disease is enriched for mesenchymal tumor cells. (a-c) IF staining for Hoechst 33258 (blue) and H2B-eGFP (green) along with luminal epithelial marker CK8 (red, top) or myoepithelial marker CK14 (red, bottom), on sections of H2B-eGFP-labeled orthotopic *HER2/neu-Prim1* (a) or *Wnt1-Prim1* (b) primary tumor (left), residual lesion 28 d after doxycycline withdrawal (right), or normal mammary gland (c). (d-g) Flow cytometry for CD49e vs. CD24 (d, e) or PDGFR-β vs. CD24 (f, g) on H2B-eGFP+DAPI- tumor cells from orthotopic H2B-eGFP-labeled orthotopic *HER2/neu-Prim1* primary tumors (d, f) or MRLs (e, g). Scale bars 200 μm for all images.

**Additional file 8: **Figure S6. HER2/neu-Prim1 residual tumor cells do not express luminal or myoepithelial markers. (a-p) IF staining for Hoechst 33258 (blue) and H2B-eGFP (green) along with epithelial markers (red) CK5 (a, i), CK18 (b, j), CK19 (c, k), EpCAM (e, m), E-Cadherin (f, n), P-Cadherin (g, o), and p63 (h, p), or with a Pan-CK antibody (d, l), on sections of MRLs from H2B-eGFP-labeled orthotopic *HER2/neu-Prim1* tumors (a-h) or normal mammary ducts (i-p). Scale bar 50 μm for all images.

**Additional file 9: **Table S3. TIC frequency for syngeneic orthotopic *MTB;TetO-HER2/neu;TTC;rYFP* primary tumors and residual lesions. Calculation of TIC frequencies for YFP+ CD45-DAPI- singlet tumor cells from syngeneic orthotopic primary tumors or residual lesions in *nu/nu* mice generated from the same *MTB;TetO-HER2/neu;TTC;rYFP* donor tumors, injected into *nu/nu* mice on doxycycline.

**Additional file 10: **Table S4. TIC frequency for syngeneic orthotopic *MTB;TetO-Wnt1;TTC;rYFP* primary tumors and residual lesions. Calculation of TIC frequencies for YFP+ CD45-DAPI- singlet tumor cells from syngeneic orthotopic primary tumors or residual lesions in *nu/nu* mice, generated from the same *MTB;TetO-Wnt1;TTC;rYFP* donor tumors, injected into *nu/nu* mice on doxycycline.

**Additional file 11: **Table S5. TIC Frequency for Tumor Cells from H2B-eGFP-labeled Orthotopic *HER2/neu-Prim1* Primary Tumors or Residual Lesions. Calculation of TIC frequencies for GFP + CD45-DAPI- singlet tumor cells from H2B-eGFP-labeled *HER2/neu-Prim1* primary tumors or residual lesions, injected into *nu/nu* mice either on (*HER2/neu* transgene expressed), or not on (*HER2/neu* transgene not expressed), doxycycline.

## Data Availability

The datasets generated and/or analyzed during the current study are available in the Gene Expression Omnibus repository, https://www.ncbi.nlm.nih.gov/gds.

## Abbreviations

BrdU: Bromodeoxyuridine
DMEM: Dulbecco’s modified Eagle’s medium
DTC: Disseminated tumor cell
ECM: Extracellular matrix
EDTA: Ethylenediaminetetraacetic acid disodium salt
EMT: Epithelial-to-mesenchymal transition
FACS: Fluorescence-activated cell sorting
FBS: Fetal bovine serum
GEM: Genetically engineered mouse
HER2/neu-Prim1: Cells from a primary *MTB;TetO-HER2/neu* tumor
HNSCC: Head and neck squamous cell carcinoma
IF: Immunofluorescence
i.v.: Intravenously
MRD: Minimal residual disease
MRL: Minimal residual lesion
MTB;TetO-HER2/neu: *MMTV-rtTA;TetO-HER2/neu*
MTB;TetO-HER2/neu;TTC;rYFP: *MTB;TetO-HER2/neu;TetO-TurboCre;Rosa26-lox-stop-lox-YFP*
MTB;TetO-Wnt: *MMTV-rtTA;TetO-Wnt1*
NSG: NOD/scid/Il2γnull
OCT: Optimal cutting temperature
O/N: Overnight
RFS: Recurrence-free survival
RT: Room temperature
TICs: Tumor-initiating cells
Wnt1-Prim1: Cells from a primary *MTB;TetO-Wnt1* tumor
