# Supplementary figures and images for: Cellular dormancy in minimal residual disease following targeted therapy
Source: Breast Cancer Res. 2021 Jun 4;23:63. doi: 10.1186/s13058-021-01416-9 (PMC8178846; doi:10.1186/s13058-021-01416-9)

Additional File 1  
Supplemental Figure 1

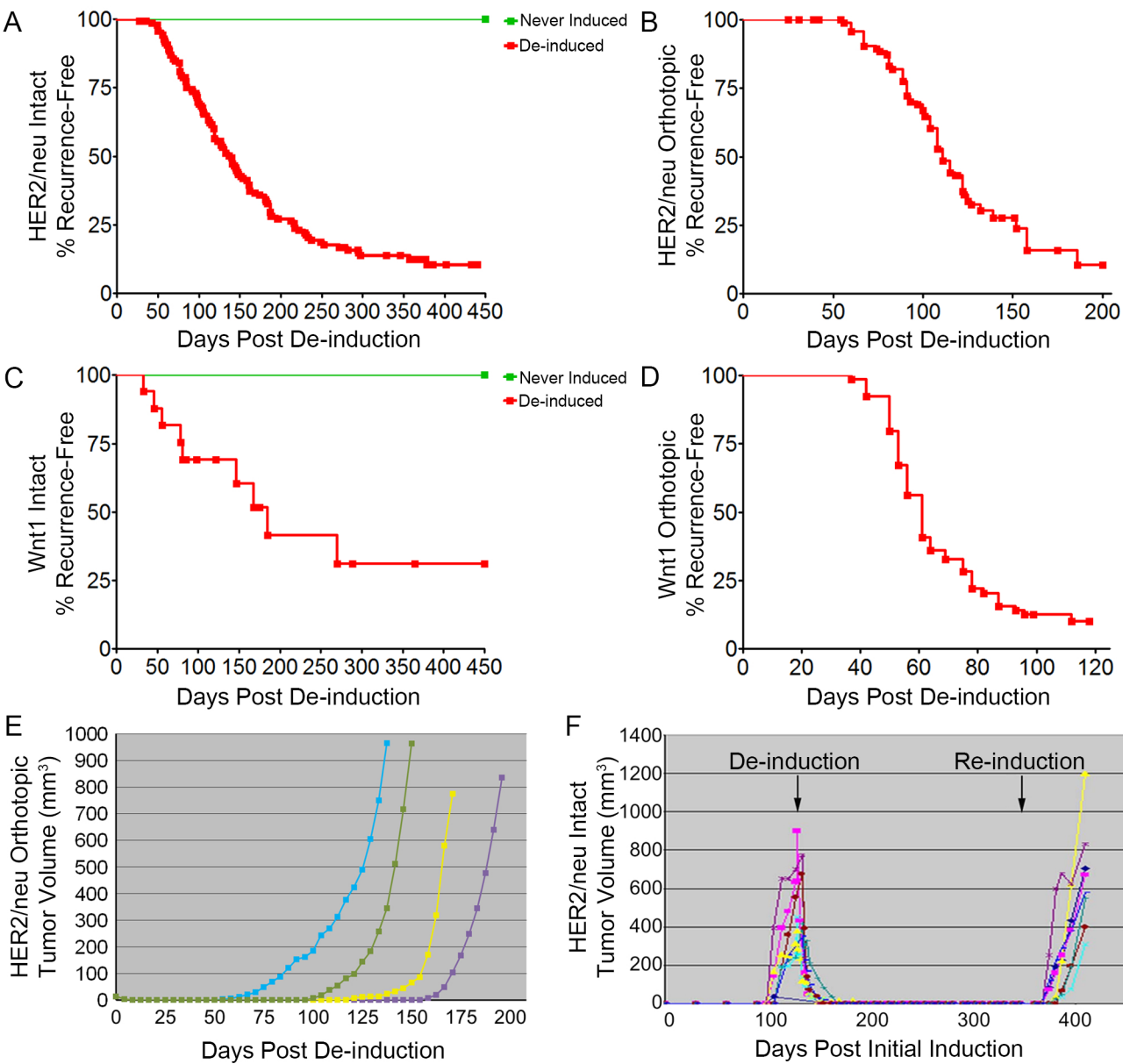

Supplement: Supplementary file 1 — Additional file 1: Figure S1. Kinetics of tumor recurrence suggest a latent phase. (a-d) Kaplan-Meier curves showing recurrence-free survival (RFS) for (a) MTB;TetO-HER2/neu intact, (b) HER2/neu-Prim1 orthotopic, (c) MTB;TetO-Wnt1 intact, and (d) Wnt1-Prim1 orthotopic models. (e) Recurrent tumor growth curves from MTB;TetO-HER2/neu orthotopic tumors with different recurrence latencies. (f) Tumor growth curves following doxycycline re-administration to intact MTB;TetO-HER2/neu mice harboring regressed primary tumors that had not spontaneously recurred. [file 13058_2021_1416_MOESM1_ESM.pdf]

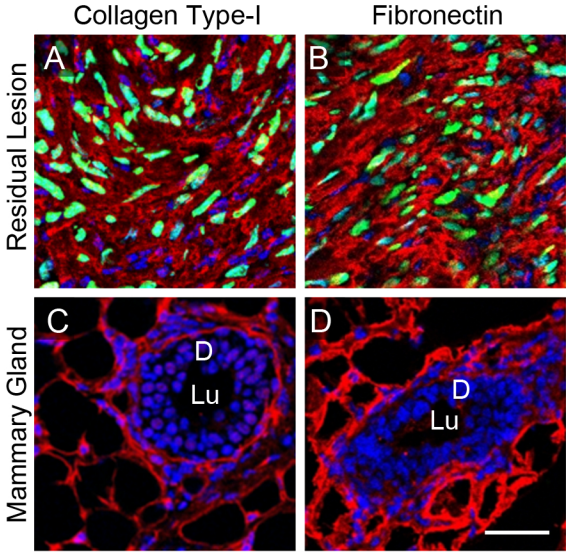

Supplement: Supplementary file 2 — Additional file 2: Figure S2. ECM protein expression in minimal residual lesions. (a-d) IF staining showing Hoechst 33258 (blue), H2B-eGFP (green) and either collagen type-I (red, a, c) or fibronectin (red, b, d) on H2B-eGFP-labeled orthotopic HER2/neu-Prim1 MRLs (a, b) or normal mammary ducts (c, d), labeled to show duct epithelial cells (D), or lumen (Lu). Scale bar 50 μm for all images. [file 13058_2021_1416_MOESM2_ESM.pdf]

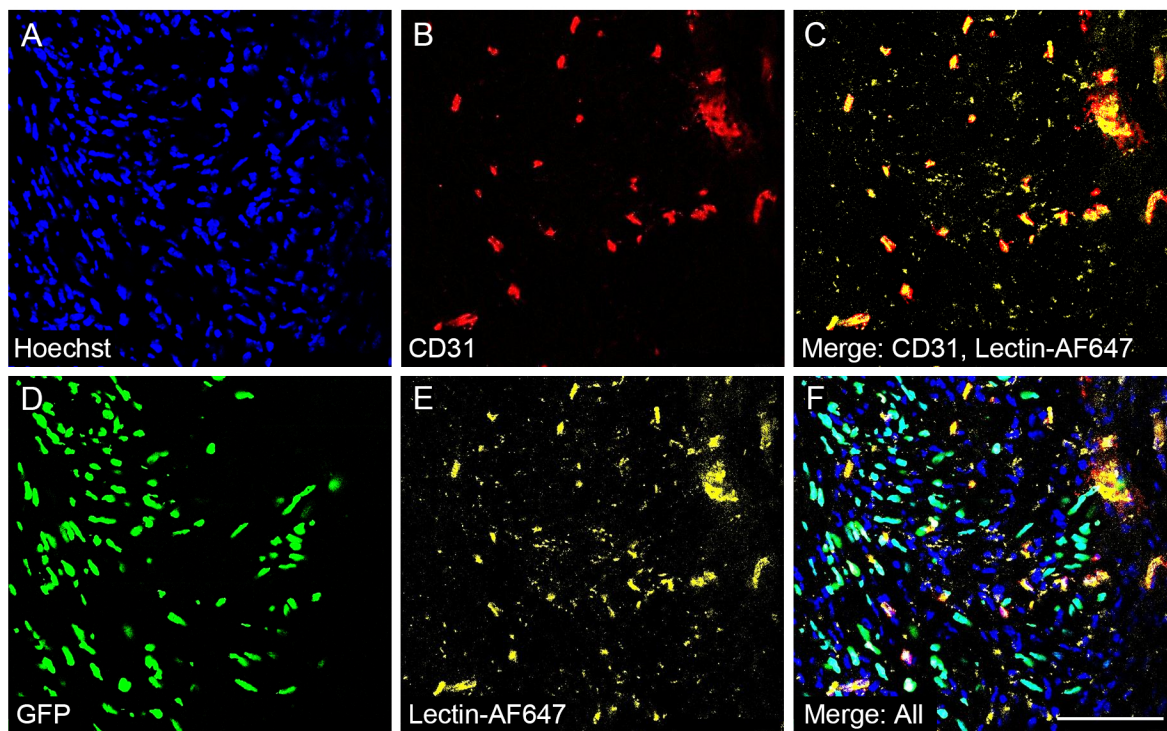

Supplement: Supplementary file 3 — Additional file 3 CD31 co-localizes with intravenously injected lectin-AF647. (a-f) Fluorescence microscopy for Hoechst 33258 (blue, a), IF for CD31 (red, b), H2B-eGFP (Green, d), lectin-AF647 (Yellow, eE), merge of CD31 and Lectin-AF647 (c), or merge of all channels (f) on H2B-eGFP-labeled orthotopic HER2/neu-Prim1 MRL. Scale bar 100 μm for all images. [file 13058_2021_1416_MOESM3_ESM.pdf]

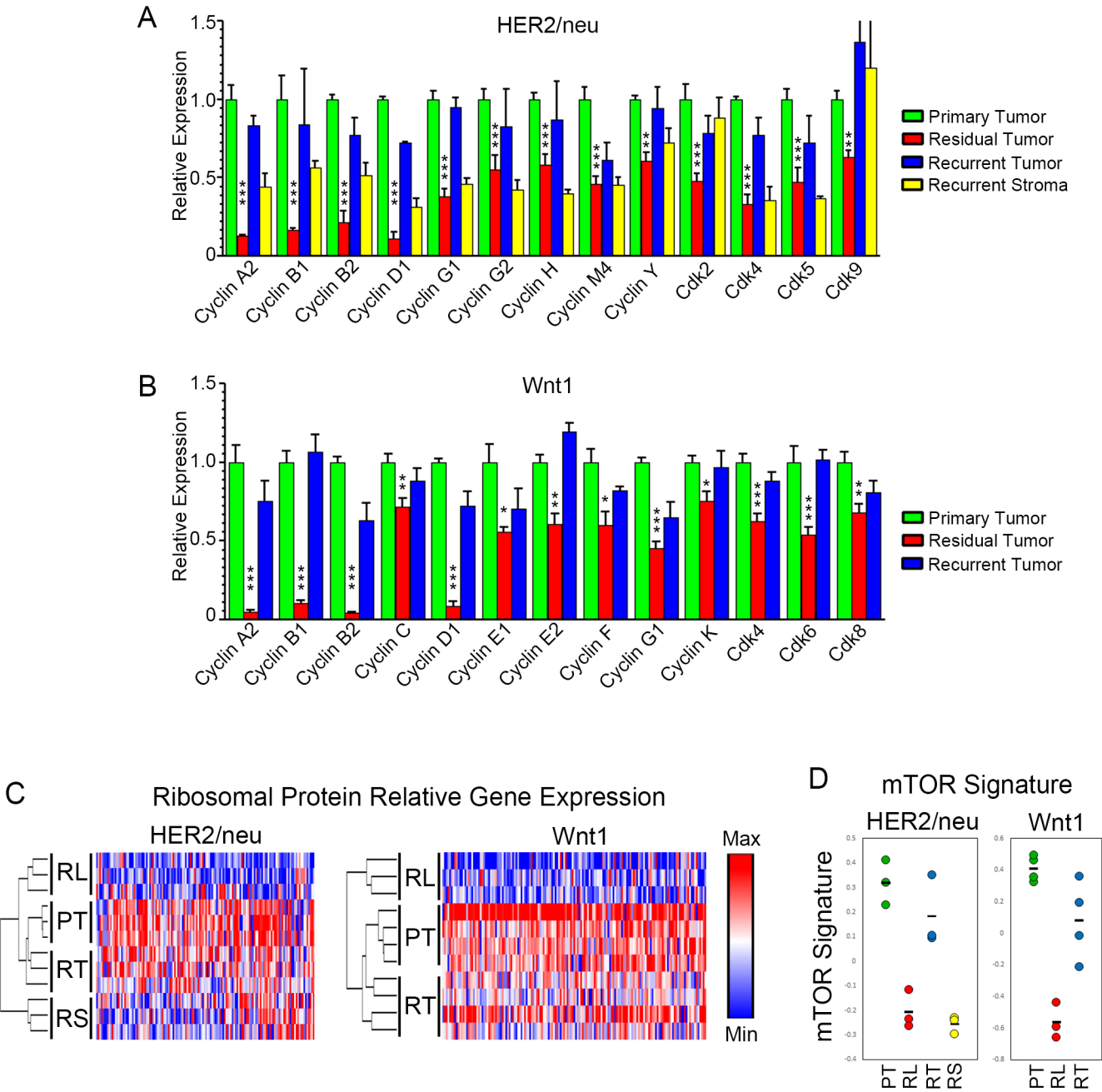

Supplement: Supplementary file 5 — Additional file 5: Figure S4. Analysis of gene expression data for mitosis-related genes and mTOR pathway activity. (a, b) Expression of all differentially expressed cyclins and cyclin-dependent kinases in HER2/neu (a) and Wnt1 (b) models. (c) Relative expression of all ribosomal proteins, and unsupervised hierarchical clustering for HER2/neu (left) and Wnt1 (right) tumors. (d) Expression of mTOR signature in primary tumor (PT), residual lesion (RL), recurrent tumor (RT) and recurrent tumor stromal cells (RS), for individual (circles) and mean (black line) of pathway activity for HER2/neu (left) and Wnt1 (right) tumors. p-value vs. RL for mTOR signature of HER2/neu: PT < 1.0E-6, RT = 2.1E-3, RS = 4.6E-3; Wnt1: PT < 1.0E-6, RT < 1.0E-4. [file 13058_2021_1416_MOESM5_ESM.pdf]

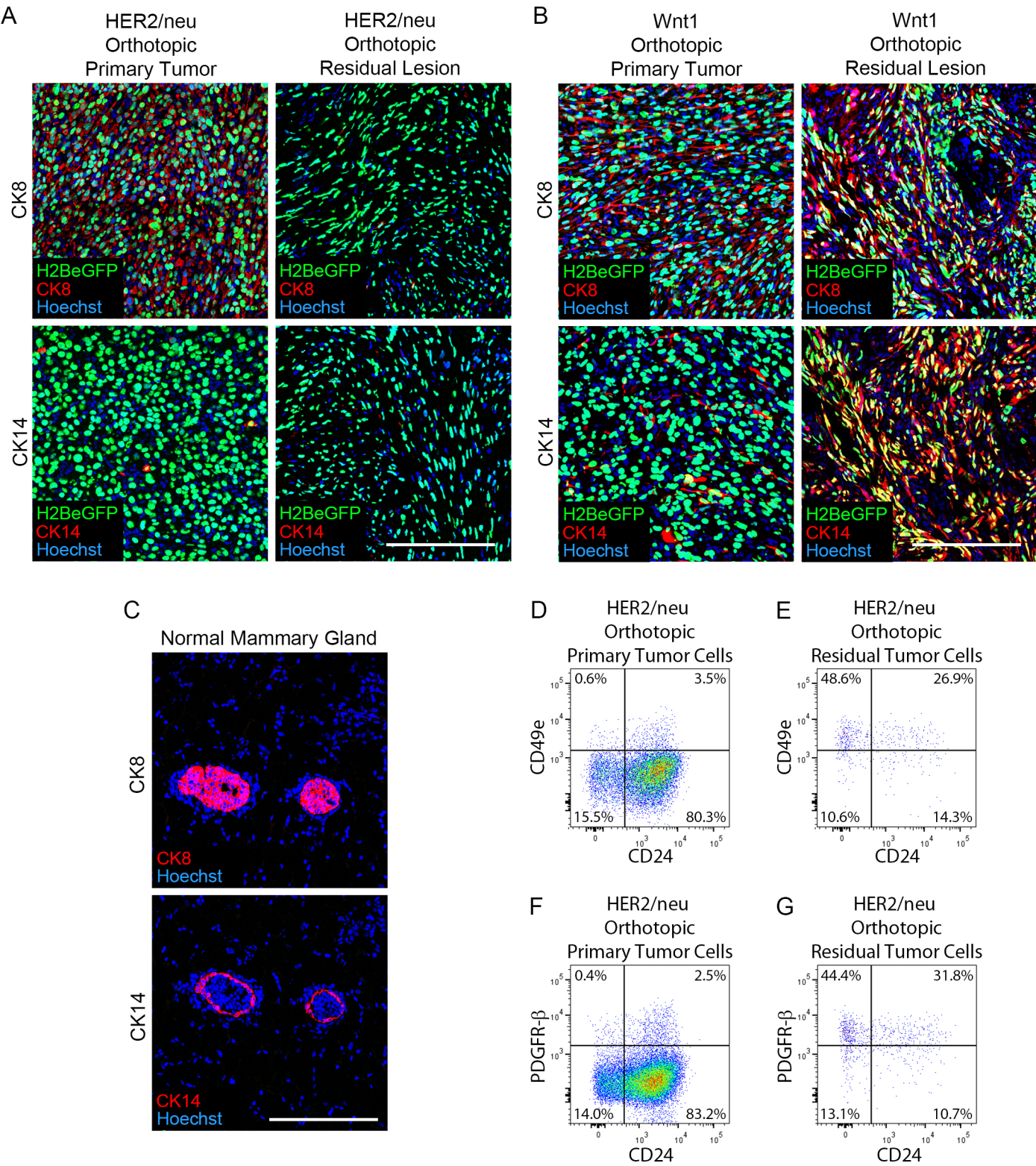

Supplement: Supplementary file 7 — Additional file 7: Figure S5. HER2/neu-Prim1 residual disease is enriched for mesenchymal tumor cells. (a-c) IF staining for Hoechst 33258 (blue) and H2B-eGFP (green) along with luminal epithelial marker CK8 (red, top) or myoepithelial marker CK14 (red, bottom), on sections of H2B-eGFP-labeled orthotopic HER2/neu-Prim1 (a) or Wnt1-Prim1 (b) primary tumor (left), residual lesion 28 d after doxycycline withdrawal (right), or normal mammary gland (c). (d-g) Flow cytometry for CD49e vs. CD24 (d, e) or PDGFR-β vs. CD24 (f, g) on H2B-eGFP+DAPI- tumor cells from orthotopic H2B-eGFP-labeled orthotopic HER2/neu-Prim1 primary tumors (d, f) or MRLs (e, g). Scale bars 200 μm for all images. [file 13058_2021_1416_MOESM7_ESM.pdf]

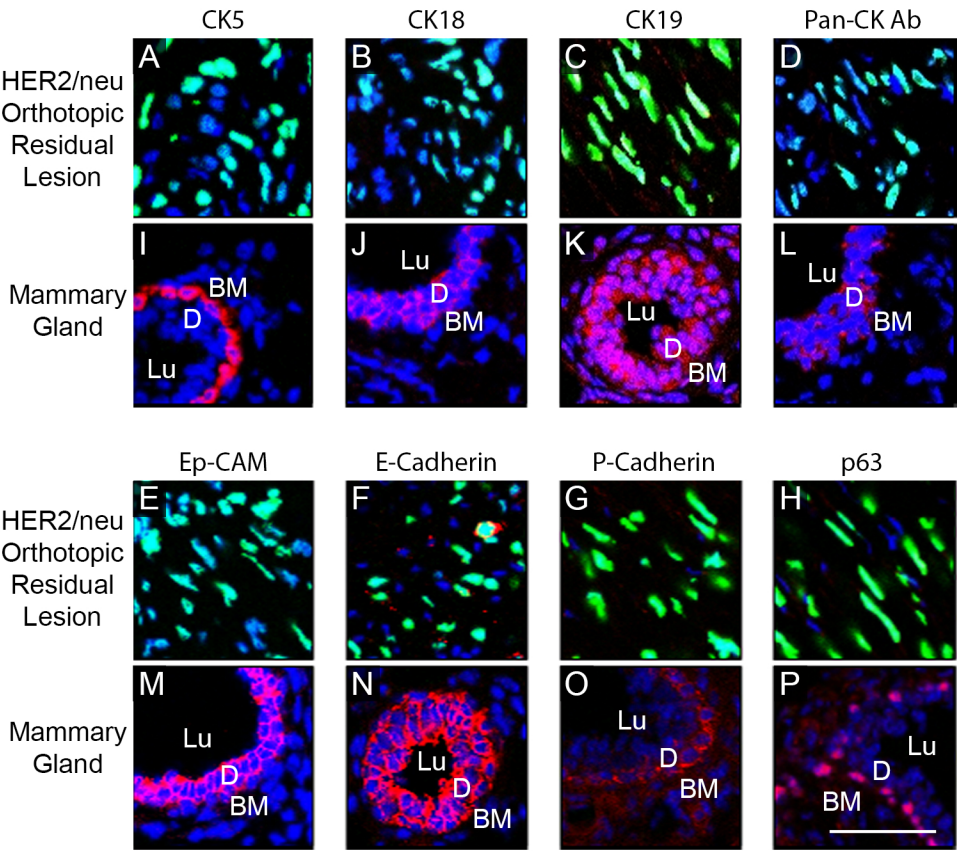

Supplement: Supplementary file 8 — Additional file 8: Figure S6. HER2/neu-Prim1 residual tumor cells do not express luminal or myoepithelial markers. (a-p) IF staining for Hoechst 33258 (blue) and H2B-eGFP (green) along with epithelial markers (red) CK5 (a, i), CK18 (b, j), CK19 (c, k), EpCAM (e, m), E-Cadherin (f, n), P-Cadherin (g, o), and p63 (h, p), or with a Pan-CK antibody (d, l), on sections of MRLs from H2B-eGFP-labeled orthotopic HER2/neu-Prim1 tumors (a-h) or normal mammary ducts (i-p). Scale bar 50 μm for all images. [file 13058_2021_1416_MOESM8_ESM.pdf]
