# Supplementary material for: Cellular dormancy in minimal residual disease following targeted therapy
Source: Breast Cancer Res. 2021 Jun 4;23:63. doi: 10.1186/s13058-021-01416-9 (PMC8178846; doi:10.1186/s13058-021-01416-9)
Supplement: Supplementary file 4 — Additional file 4:. Table S1. Gene sets down-regulated in dormant residual tumor cells. Clusters of SP-PIR Keywords identified by DAVID functional ontology analysis of genes down-regulated in dormant residual tumor cells compared to all other cell types (false discovery rate (FDR) < 0.1, fold-change (FC) > 1.5-fold). [file 13058_2021_1416_MOESM4_ESM.pdf]

Additional File 4
Table S1

Gene Sets Down-regulated in Wnt1 Dormant Residual Tumor Cells

	SP_PIR_KEYWORDS	# Down-regulated Genes in Cluster	p-value	FDR (Benjamini)
Cluster 1	Enrichment Score: 38.87			
	cell cycle	132	1.80E-49	4.30E-47
	mitosis	74	1.10E-38	1.40E-36
	cell division	88	1.50E-38	1.50E-36
Cluster 2	Enrichment Score: 24.81			
	kinetochore	36	1.60E-25	1.10E-23
	chromosomal protein	42	1.00E-16	4.10E-15
Cluster 3	Enrichment Score: 17.26			
	ribonucleoprotein	71	1.00E-23	6.10E-22
	ribosomal protein	52	1.70E-19	9.20E-18
Cluster 4	Enrichment Score: 16.28			
	dna replication	34	2.00E-17	8.90E-16
Cluster 6	Enrichment Score: 11.13			
	DNA damage	38	8.40E-09	2.30E-07
	dna repair	34	5.30E-08	1.40E-06
Cluster 7	Enrichment Score: 11			
	atp-binding	152	9.10E-12	2.90E-10
	nucleotide-binding	176	3.00E-10	9.10E-09
Cluster 8	Enrichment Score: 7.67			
	ubl conjugation	83	7.20E-13	2.50E-11
	isopeptide bond	42	1.90E-06	3.80E-05
Cluster 9	Enrichment Score: 6.33			
	ribosome biogenesis	15	9.10E-07	1.90E-05
	rrna processing	15	3.00E-05	5.00E-04
Cluster 10	Enrichment Score: 6.03			
	cytoskeleton	71	2.00E-06	4.00E-05
	microtubule	34	1.50E-05	2.80E-04
Cluster 12	Enrichment Score: 5.62			
	chromosomal protein	42	1.00E-16	4.10E-15
Cluster 15	Enrichment Score: 4.79			
	molecular chaperone	7	1.80E-04	2.50E-03
Cluster 20	Enrichment Score: 3.52			
	mitochondrion	78	7.80E-04	1.00E-02
	transit peptide	47	4.60E-03	4.40E-02

Gene Sets Down-regulated in HER2/neu Dormant Residual Tumor Cells

	SP_PIR_KEYWORDS	# Down-regulated Genes in Cluster	p-value	FDR (Benjamini)
Cluster 1	Enrichment Score: 25.71			
	ribonucleoprotein	85	3.90E-31	6.20E-29
	ribosomal protein	67	1.70E-29	2.10E-27
Cluster 2	Enrichment Score: 24.79			
	cell division	79	7.80E-28	7.40E-26
	cell cycle	107	3.10E-27	2.40E-25
	mitosis	62	6.50E-25	3.40E-23
Cluster 4	Enrichment Score: 12.94			
	mitochondrion	149	4.70E-26	2.80E-24
	transit peptide	85	1.50E-14	5.80E-13
Cluster 5	Enrichment Score: 11.29			
	chromosomal protein	33	5.30E-09	1.30E-07
Cluster 6	Enrichment Score: 9.67			
	kinetochore	26	4.40E-13	1.60E-11
Cluster 7	Enrichment Score: 9.26			
	ubl conjugation	88	1.90E-12	6.40E-11
	isopeptide bond	51	6.30E-09	1.40E-07
Cluster 8	Enrichment Score: 9.1			
	rrna processing	22	7.50E-10	2.10E-08
	ribosome biogenesis	16	5.40E-07	9.50E-06
Cluster 9	Enrichment Score: 7.04			
	dna replication	27	3.40E-10	1.00E-08
Cluster 10	Enrichment Score: 6.69			
	nucleotide-binding	189	1.20E-09	3.30E-08
	atp-binding	152	2.10E-08	4.10E-07
Cluster 11	Enrichment Score: 6.44			
	dna repair	33	1.90E-06	3.00E-05
	DNA damage	35	3.20E-06	4.60E-05
Cluster 14	Enrichment Score: 5.85			
	cytoskeleton	67	6.10E-04	6.30E-03
Cluster 19	Enrichment Score: 4.99			
	molecular chaperone	6	2.70E-03	2.30E-02
Cluster 25	Enrichment Score: 3.72			
	microtubule	28	9.00E-03	6.70E-02
