# Supplementary material for: Cellular dormancy in minimal residual disease following targeted therapy
Source: Breast Cancer Res. 2021 Jun 4;23:63. doi: 10.1186/s13058-021-01416-9 (PMC8178846; doi:10.1186/s13058-021-01416-9)
Supplement: Supplementary file 6 — Additional file 6:. Table S2. Gene sets up-regulated in dormant residual tumor cells. Clusters of SP-PIR Keywords identified by DAVID functional ontology analysis of genes up-regulated in dormant residual tumor cells compared to all other cell types (FDR < 0.1, FC > 1.5-fold). [file 13058_2021_1416_MOESM6_ESM.pdf]

Additional File 6
Table S2

Gene Sets Up-regulated in Wnt1 Dormant Residual Tumor Cells

	SP_PIR_KEYWORDS	# Up-regulated Genes in Cluster	p-value	FDR (Benjamini)
Cluster 1	Enrichment Score: 26.84			
	signal	276	8.20E-36	3.20E-33
	glycoprotein	302	2.00E-31	4.00E-29
	Secreted	151	6.80E-24	9.00E-22
	disulfide bond	211	1.50E-21	1.20E-19
Cluster 2	Enrichment Score: 15.65			
	extracellular matrix	42	2.00E-15	1.30E-13
Cluster 3	Enrichment Score: 14.27			
	cell adhesion	67	1.20E-21	1.20E-19
Cluster 4	Enrichment Score: 6.43			
	membrane	331	2.40E-10	1.40E-08
	transmembrane	305	7.70E-08	3.40E-06
Cluster 5	Enrichment Score: 4.94			
	heparin-binding	11	1.10E-04	3.10E-03

Gene Sets Up-regulated in HER2/neu Dormant Residual Tumor Cells

	SP_PIR_KEYWORDS	# Up-regulated Genes in Cluster	p-value	FDR (Benjamini)
Cluster 1	Enrichment Score: 12.94			
	signal	163	2.60E-16	8.00E-14
	glycoprotein	186	2.80E-16	6.00E-14
	Secreted	92	9.90E-13	1.20E-10
	disulfide bond	124	1.50E-09	1.30E-07
Cluster 2	Enrichment Score: 5.93			
	extracellular matrix	21	5.30E-06	3.10E-04
Cluster 3	Enrichment Score: 5.75			
	cell adhesion	33	1.10E-07	7.60E-06
Cluster 6	Enrichment Score: 2.83			
	heparin-binding	7	4.50E-03	1.50E-01
Cluster 7	Enrichment Score: 2.31			
	membrane	196	1.20E-03	5.00E-02
	transmembrane	184	3.40E-03	1.30E-01
