# Supplementary material for: Cellular dormancy in minimal residual disease following targeted therapy
Source: Breast Cancer Res. 2021 Jun 4;23:63. doi: 10.1186/s13058-021-01416-9 (PMC8178846; doi:10.1186/s13058-021-01416-9)
Supplement: Supplementary file 9 — Additional file 9: Table S3. TIC frequency for syngeneic orthotopic MTB;TetO-HER2/neu;TTC;rYFP primary tumors and residual lesions. Calculation of TIC frequencies for YFP+ CD45-DAPI- singlet tumor cells from syngeneic orthotopic primary tumors or residual lesions in nu/nu mice generated from the same MTB;TetO-HER2/neu;TTC;rYFP donor tumors, injected into nu/nu mice on doxycycline. [file 13058_2021_1416_MOESM9_ESM.pdf]

Additional File 9
Table S3

HER2/neu Donor Tumor Type	HER2/neu Orthotopic Tumor Type	# Tumor Cells Injected				TIC frequency	95%CI Upper Limit	95%CI Lower Limit
		500	50	5	1			
Donor Tumor A	Primary Tumor A	1/8	0/8	0/8	0/8	1 in 4,193	1 in 593	1 in 29,638
	Residual Lesion A	1/8	0/8	0/8	0/8	1 in 4,193	1 in 593	1 in 29,638
Donor Tumor B	Primary Tumor B	8/8	8/8	6/8	1/8	1 in 4	1 in 2	1 in 9
	Residual Lesion B	1/8	0/8	0/8	0/8	1 in 4,193	1 in 593	1 in 29,638
Donor Tumor C	Primary Tumor C	8/8	8/8	0/8	0/8	1 in 22	1 in 11	1 in 48
	Residual Lesion C	NA	2/16	0/8	0/8	1 in 398	1 in 100	1 in 1,591
