# Supplementary material for: Cellular dormancy in minimal residual disease following targeted therapy
Source: Breast Cancer Res. 2021 Jun 4;23:63. doi: 10.1186/s13058-021-01416-9 (PMC8178846; doi:10.1186/s13058-021-01416-9)
Supplement: Supplementary file 10 — Additional file 10: Table S4. TIC frequency for syngeneic orthotopic MTB;TetO-Wnt1;TTC;rYFP primary tumors and residual lesions. Calculation of TIC frequencies for YFP+ CD45-DAPI- singlet tumor cells from syngeneic orthotopic primary tumors or residual lesions in nu/nu mice, generated from the same MTB;TetO-Wnt1;TTC;rYFP donor tumors, injected into nu/nu mice on doxycycline. [file 13058_2021_1416_MOESM10_ESM.pdf]

Additional File 10
Table S4

HER2/neu Donor Tumor Type	HER2/neu Orthotopic Tumor Type	# Tumor Cells Injected					TIC frequency	95%CI Upper Limit	95%CI Lower Limit
		50,000	10,000	1,000	100	10			
Donor Tumor A	Primary Tumor A	7/8	4/8	0/8	0/8	0/8	1 in 20,717	1 in 10,363	1 in 41,420
	Residual Lesion A	2/8	0/8	0/8	0/8	0/8	1 in 218,487	1 in 55,163	1 in 865,380
Donor Tumor B	Primary Tumor B	6/8	2/10	0/10	0/10	0/12	1 in 40,033	1 in 19,563	1 in 81,923
	Residual Lesion B	1/4	0/10	0/10	0/10	0/12	1 in 285,390	1 in 41,155	1 in1,979,066
