# Supplementary material for: Cellular dormancy in minimal residual disease following targeted therapy
Source: Breast Cancer Res. 2021 Jun 4;23:63. doi: 10.1186/s13058-021-01416-9 (PMC8178846; doi:10.1186/s13058-021-01416-9)
Supplement: Supplementary file 11 — Additional file 11: Table S5. TIC Frequency for Tumor Cells from H2B-eGFP-labeled Orthotopic HER2/neu-Prim1 Primary Tumors or Residual Lesions. Calculation of TIC frequencies for GFP + CD45-DAPI- singlet tumor cells from H2B-eGFP-labeled HER2/neu-Prim1 primary tumors or residual lesions, injected into nu/nu mice either on (HER2/neu transgene expressed), or not on (HER2/neu transgene not expressed), doxycycline. [file 13058_2021_1416_MOESM11_ESM.pdf]

Additional File 11
Table S5

HER2/neu-Prim1 H2BeGFP Donor Tumor Type	*nu/nu* recipient on Doxycycline?	# Tumor Cells			TIC frequency	95%CI Upper Limit	95%CI Lower Limit
		500	50	5			
Primary Tumor	Yes	6/6	6/6	5/6	1 in 3	1 in 1	1 in 8
Residual Lesion	Yes	6/6	6/6	3/5	1 in 5	1 in 2	1 in 17
Primary Tumor	No	0/6	0/6	0/6	< 1 in 3,073		
Residual Lesion	No	4/6	0/6	1/6	1 in 427	1 in 168	1 in 1,085
